# Morpholino-driven blockade of Dkk-1 in osteosarcoma inhibits bone damage and tumour expansion by multiple mechanisms

**DOI:** 10.1038/s41416-022-01764-z

**Published:** 2022-03-11

**Authors:** Simin Pan, Michael Cesarek, Carla Godoy, Cynthia M. Co, Catherine Schindler, Kelbi Padilla, Andrew Haskell, Heather Barreda, Christopher Story, Roy Poole, Alan Dabney, Carl A. Gregory

**Affiliations:** 1 Department of Molecular and Cellular Medicine, Institute for Regenerative Medicine, Texas A&M Health Science Center, Bryan, TX 77807 USA; 2 Department of Statistics, Texas A&M University, College Station, TX 77843 USA; 3 Department of Veterinary Pathobiology, Texas A&M College of Veterinary Medicine and Biomedical Sciences, College Station, TX 77843 USA

**Keywords:** Bone cancer, Target validation

## Abstract

**Background:**

Osteosarcoma (OS) is the most common primary bone malignancy. Chemotherapy plays an essential role in OS treatment, potentially doubling 5-year event-free survival if tumour necrosis can be stimulated. The canonical Wnt inhibitor Dickkopf-1 (Dkk-1) enhances OS survival in part through upregulation of aldehyde-dehydrogenase-1A1 which neutralises reactive oxygen species originating from nutritional stress and chemotherapeutic challenge.

**Methods:**

A vivo morpholino (DkkMo) was employed to block the expression of Dkk-1 in OS cells. Cell mitosis, gene expression and bone destruction were measured in vitro and in vivo in the presence and absence of doxorubicin (DRB).

**Results:**

DkkMo reduced the expression of Dkk-1 and Aldh1a1, reduced expansion of OS tumours, preserved bone volume and architecture and stimulated tumour necrosis. This was observed in the presence or absence of DRB.

**Conclusion:**

These results indicate that administration of DkkMo with or without chemotherapeutics can substantially improve OS outcome with respect to tumour expansion and osteolytic corruption of bone in experimental OS model.

## Background

Osteosarcoma (OS) is the most common primary bone malignancy. Particularly common in paediatric patients, it accounts for ~9% of paediatric cancer deaths [1]. The current standard of care for treating OS is surgery with chemotherapy [2]. Chemotherapeutic strategies for treating osteosarcoma generally include methotrexate, doxorubicin (DRB) and cisplatin [2]. These agents play a positive role in OS treatment, with a reported increase in 5-year event-free survival from 20–40% (surgery alone) to 50–90% with the successful chemotherapeutic intervention [3]. These survival rates can be stratified into responders with greater than 90% tumour necrosis after neoadjuvant therapy versus the remainder, who have 5-year event-free survival rates of 90% and 50–60%, respectively [3]. In spite of the benefits, long-term chemotherapy results in side effects that can be catastrophic to patients’ health and quality of life [4–6]. Many patients also experience pain and immobility as a result of osteolytic bone lesions (OLs), which occur due to excessive bone resorption [7]. OLs not only cause significant pain but also increase the risk of fracture and contribute to the vicious cycle between cancer cells, osteoblasts and osteoclasts which provides the ideal environment for tumour propagation [8]. Reducing the dependence on chemotherapy and the OL burden would significantly improve the impact of OS treatment strategies.

High expression of Dickkopf–Wnt-signalling pathway inhibitor-1 (Dkk-1) occurs in various cancers, including multiple myeloma and OS [9–14]. Our previous study found that constitutively high expression of human Dkk-1 in the OS cell line MOSJ-Dkk-1 increases tumour growth rate and bone destruction in mouse models when compared to control lines which manifested tumours primarily as non-osteolytic chondroblastic OS nodules [15]. The observed increase in proliferation and tumorigenicity was found to be due in part to a stress response modulated by enhanced expression of aldehyde-dehydrogenase-1A1 (Aldh1a1). This occurred through inhibition of canonical Wnt signalling (cWnt) by Dkk-1, driving the balance of Wnt signalling in favour of a non-canonical Wnt pathway (ncWnt) which upregulated Aldh1a1 expression through activation of Jun kinase (JNK). Aldh1a1 is a known member of the stress response arsenal, neutralising free radicals from metabolic stressors and chemotherapeutics [15]. The aldehyde-dehydrogenase family has also been implicated as a major driver of chemoresistance and survival in cancer stem cells [16]. Dkk-1 also inhibits cWnt-mediated differentiation of osteoblasts thereby preventing the repair of OLs [12, 15]. Therefore, targeting Dkk-1 in OS tumours could reduce the expansion and survival of tumour cells, increase susceptibility to chemotherapeutics, and restore the capacity of bone to repair itself.

Herein, we demonstrate that through inhibiting Dkk-1 transcription by means of a vivo morpholino (DkkMo), it is possible to (i) reduce the expansion of MOSJ-Dkk-1 tumours in vitro and vivo, (ii) preserve bone volume and architecture in vivo and (iii) stimulate necrosis of the tumour. DkkMo had the capacity to perform these functions in the presence or absence of DRB, and the level of tumour growth inhibition by DkkMo when administered as a single agent was equivalent to that observed by DRB at a high dose. In contrast with DRB, DkkMo did not cause weight loss in mice. RNA sequencing indicated that DkkMo stimulated cell death and necrotic mechanisms in tumours. Collectively, these results indicate that administration of DkkMo in the presence or absence of chemotherapeutics has the capacity to substantially improve outcomes with respect to OS tumour expansion and osteolytic corruption of bone.

## Methods

### Detailed methods are provided in the Supplemental Materials

#### Tissue culture

MOSJ-pLenti cells were generated and cultured as previously described [15].

#### In vivo model of osteolytic OS

Vertebrate animals were utilised in accordance with a protocol approved by the Texas A&M Institutional Animal Care and Use Committee. MOSJ-Dkk-1 cells were employed in the OS xenograft model as described [15].

#### Bone deformation

3D Slicer software was used for bone volume and deformation measurements. Three-dimensional renderings of the tumour-bearing and contralateral fibulae were constructed using the Otsu thresholding method. Model to model distances was calculated with the signed closest point approach.

#### High-throughput RNA sequencing

Samples are sequenced by BGI Genomics (BGI Americas Corp. Cambridge, MA 02142, USA).

#### Neural net programming and utilisation

A U-net architecture based Convolutional Neural Network (CNN) was built using TensorFlow v.2.3.0 [17, 18].

#### Statistics

GraphPad Prism version 8.00 and R for Mac was used for statistical analysis.

## Results

### Blockade of Dkk-1 with a vivo morpholino

Previously we demonstrated that expression of human Dkk-1 by the chondroblastic OS sarcoma cell line MOSJ [19] (MOSJ-Dkk-1 cells) resulted in accelerated tumour growth and an aggressive osteolytic phenotype [15]. Given the role of Dkk-1 in tumour propagation and bone destruction in malignant bone diseases, we hypothesised that blockade of Dkk-1 could have a dual role in the inhibition of bone destruction and tumour expansion. Immunoblockade of Dkk-1 has been successfully achieved in multiple myeloma [20] and OS [21], but systemic Dkk-1 levels can be substantial [10, 12], requiring large doses of antibodies. As such, blockade of Dkk-1 transcription may represent a more efficient approach.

**Fig. 1:**
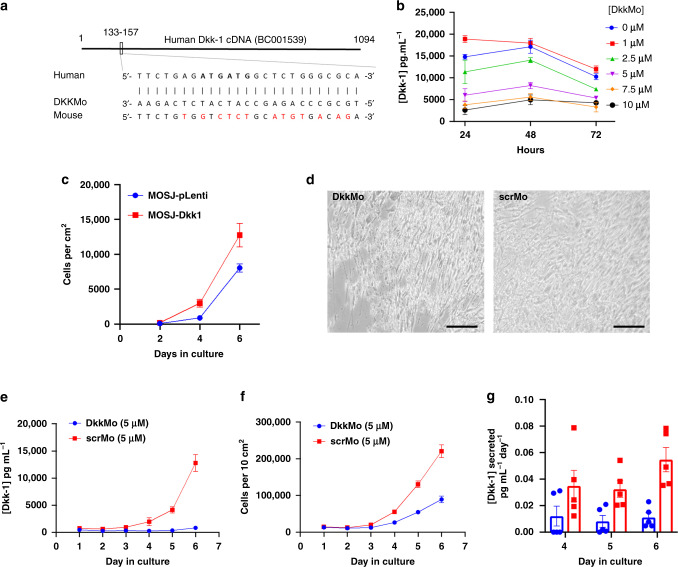
DkkMo blocks Dkk-1 expression by MOSJ-Dkk-1 cells resulting in inhibition of expansion in vitro. **a** Target sequence of DkkMo, position on the Dkk-1 mRNA and comparison with the mouse orthologue. **b** DkkMo dose-dependently reduces the secretion of Dkk-1 by MOSJ-Dkk-1 cells (*n* = 4). **c** Expansion of MOSJ-Dkk-1 cells compared to control MOSJ-pLenti cells that do not express Dkk-1 (*n* = 3). **d** DkkMo inhibits resistance to nutritional stress caused by extended durations of confluent culture. **e** Dkk-1 secretion by MOSJ-Dkk-1 cells in the presence of DkkMo or scrMo during expansion in culture (*n* = 5). **f** Cell expansion in the presence of DkkMo or scrMo over time in culture (*n* = 5). **g** Dkk-1 secretion normalised to cell number (legend as panels **e** and **f**).

To facilitate in vivo delivery and biodistribution of the blocking nucleotide, vivo morpholino technology was chosen, and a custom vivo morpholino targeting the 25 nucleotides at the 5’ end of the cDNA was designed by Gene tools LLC, hereafter referred to as DkkMo (Fig. 1a). As a control, a vivo morpholino was designed with five mismatches (scrMo). DkkMo (but not scrMo) dose-dependently reduced secretion of Dkk-1 by MOSJ-Dkk-1 cells (Fig. 1b). In contrast with MOSJ-Dkk-1 cells, MOSJ-pLenti cells that do not express high levels of Dkk-1 have a slower rate of proliferation (Fig. 1c) and are sensitive to confluence and nutritional stressors [15]. MOSJ-Dkk-1 cells survive for extended durations under these conditions, but exposure to DkkMo negated this capability (Fig. 1d). Loss of Dkk-1 through the addition of DkkMo also reduced the rate of proliferation of MOSJ-Dkk-1 cells (Fig. 1e, f). The normalisation of Dkk-1 to cell number confirmed that reduced cell counts did not account for the reduced Dkk-1 output (Fig. 1g).

### DkkMo reduces Aldh1a1 production and disrupts stress response and survival pathways

**Fig. 2:**
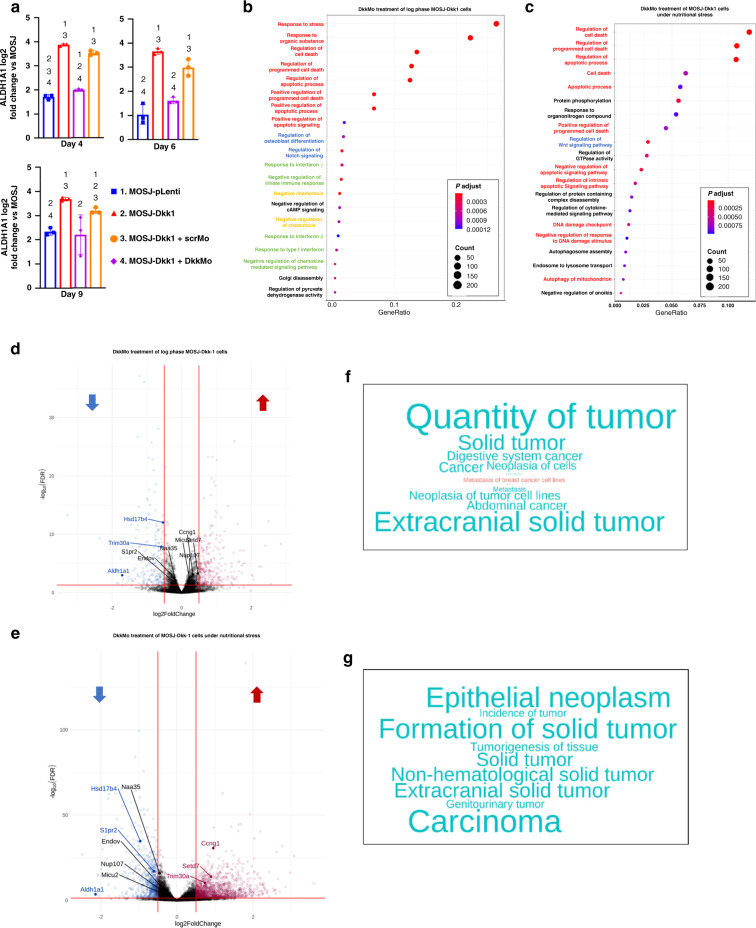
DkkMo blocks Aldh1a1 transcription, stress response and cell expansion pathways in MOSJ-Dkk-1 cells in vitro. **a** Transcription of Aldh1a1 as measured by qRT-PCR by MOSJ-Dkk-1 cells in the presence of DkkMo and scrMo (*n* = 3). Numbering over a given measurement indicates those conditions where measurements differ with *P*adj < = 0.05. **b** Gene ontology term enrichment analysis of RNA sequencing data with a comparison between DkkMo and scrMo treatment of MOSJ-Dkk-1 cells undergoing rapid proliferation (*n* = 3). Red font refers to gene ontologies that relate to cell death and survival, blue refers to osteogenic differentiation, green refers to immunoregulation and yellow refers to chemotaxis. **c** As panel **b**, but under extended durations of nutritional stress. **d** Volcano plot of DE genes after comparison of MOSJ-Dkk-1 cells at log phase of growth in presence of DkkMo (10 μM) or scrMo. Sequences that represent both DE and hub genes are labelled. **e** As panel **d**, but with MOSJ-Dkk-1 cells subjected to extended confluence under nutritional stress. **f** Word cloud summarising IPA results using DE gene lists from the comparison of MOSJ-Dkk-1 cells at log phase of growth in presence of DkkMo or scrMo. The words represent functional categories, the size of the font represents the size of the group, red and blue font represents upregulated and downregulated sequences respectively. **g** As panel **f**, but with MOSJ-Dkk-1 cells subjected to extended confluence under nutritional stress.

In MOSJ-Dkk-1 OS cells, Dkk-1 triggers a stress response pathway by inhibition of cWnt signalling, activating a ncWnt pathway that facilitates JNK/Jun mediated expression of Aldh1a1 [15]. Aldh1a1 is one of the major aldehyde dehydrogenases (ALDH) responsible for the neutralisation of reactive oxygen species (ROS) that occur when cells are under nutritional and chemotherapeutic stress [22–25], and Aldh1a1 enhances chemoresistance, metastasis and tumour initiator capability [22, 26–29]. It was therefore hypothesised that inhibition of Dkk-1 with DkkMo may increase susceptibility to nutritional and chemotherapeutic stressors by reducing the expression of Aldh1a1. To test the effect of DkkMo on Aldh1a1 expression, MOSJ-Dkk-1 cells were exposed to 5 μM DkkMo for 4, 6 and 9 days and Aldh1a1 transcription was assayed by quantitative RT-PCR (qRT-PCR). At each timepoint, DkkMo reduced Aldh1a1 transcription to levels observed in MOSJ-pLenti cells (Fig. 2a).

To gain broader insight into the effects of Dkk-1 blockade, MOSJ-Dkk-1 cells at logarithmic and confluent phases of growth were exposed to DkkMo for 6 days and mRNA was recovered for high-throughput RNA sequencing (HTS). The rationale for these culture conditions was to induce stress through mitosis or starvation, respectively. Differentially expressed (DE) sequences (by > 1.5-fold) between DkkMo-treated and scrMo-treated cultures were (Supplemental Table 1) categorised based on gene ontology (GO) term enrichment analysis. The DE lists were first compared to genes known to be upregulated by cWnt signalling curated by the Nusse group [https://web.stanford.edu/group/nusselab/cgi-bin/wnt/target_genes] to examine whether DkkMo had the expected effect of upregulating genes known to be activated by cWnt. For MOSJ-Dkk-1 cells in the log phase of growth, genes upregulated by DkkMo treatment shared composition with genes known to be upregulated by cWnt signalling with a false discovery-rate-adjusted *P* value of < = 0.018 whereas the list of downregulated genes compared to the same list was not significant (*P*adj > 0.5). These results indicate that the result of blocking Dkk-1 by DkkMo results in the predictable outcome of upregulation of cWnt signalling. DE genes upregulated under confluent conditions did not exhibit the same degree of similarity to Wnt upregulated genes, presumably due to noise attributed to massive upregulation of apoptotic and stress pathways that occurred with the starvation of the confluent monolayers.

In both culture conditions, the greatest degree of DE gene enrichment occurred in GO-term lists related to stress response, programmed cell death, and response to chemical stimuli (Fig. 2b, c). In the case of rapidly dividing cells, GO terms related to chemotaxis, osteogenesis and immune-chemokines were also represented (Fig. 2b). Co-expressed, functionally related gene modules were also calculated from the dataset and hub genes with the highest degree of connectivity within each module identified (Supplementary Fig. S2). Hub genes were plotted on volcano plots to visualize potential overlap between lists of DE genes (Fig. 2d, e). In response to DkkMo, Aldh1a1 was downregulated in both culture conditions and was identified as a hub gene, further supporting the close relationship between Dkk-1 and Aldh1a1. Hsd17b4, encoding 17β-hydroxysteroid dehydrogenase/D-3-hydroxy acyl-CoA dehydrogenase also met these criteria [30]. Dkk-1 did not appear in DE gene lists because the ectopically expressed Dkk-1 was of human origin and comparisons were made against a murine reference genome. Ingenuity Pathway Analysis (IPA) was performed on DE datasets to identify differentially regulated processes in response to Dkk-1. For both culture conditions, DkkMo treatment downregulated genes involved in tumour expansion (Fig. 2f, g). These data indicate that Dkk-1 blockade by DkkMo disrupts mitotic and survival pathways involved in the expansion of MOSJ-Dkk-1 cells and provides strong support for the role of Aldh1a1 in this process.

#### Dkk-1 desensitises MOSJ cells to DRB

**Fig. 3:**
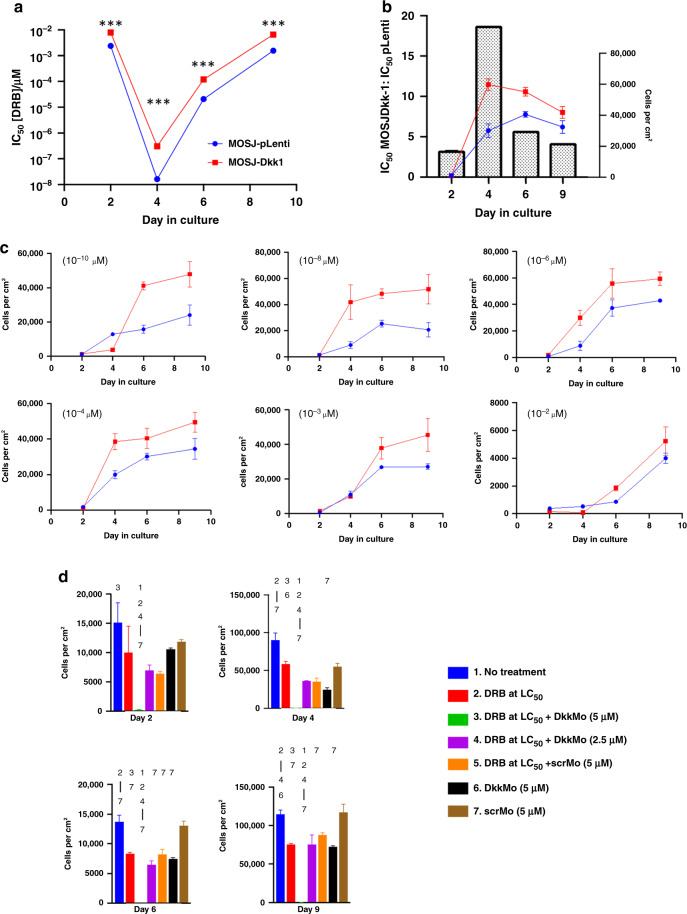
Dkk-1 enhances resistance to challenge with DRB. **a** IC_50_ calculations on MOSJ-Dkk-1 and MOSJ-pLenti cells treated with various doses of DRB by time in culture. **b** Plot (bars) of the ratio of IC_50_ MOSJ-Dkk-1 cells to MOSJ-pLenti cells when compared to the rate of expansion (lines). The greatest difference between the IC_50_ values occurs during the highest rate of cell division. **c** Growth curves of MOSK-Dkk-1 cells and MOSJ-pLenti cells in the presence of various concentrations of DRB. **d** MOSJ-Dkk-1 cell yields after treatment with DkkMo, scrMo, DRB or combinations. Numbering refers to conditions where measurements differ with *P*adj < = 0.05. *P*-values were calculated by the ratio-based test against a change in persistence method [70].

DRB is used for the treatment of OS [3], and chemoresistance to DRB in OS has been attributed to elevated ALDH activity [22, 31]. This observation is not surprising, given that one mechanism of DRB is the generation of ROS which destroys tumour cells [32]. We hypothesised that Dkk-1 expression and associated Aldh1a1 levels could increase resistance to DRB, and the blockade of Dkk-1 by DkkMo could reverse this. Identical cultures of rapidly expanding MOSJ-Dkk-1 and MOSJ-pLenti cells were exposed to DRB at doses ranging from 10^−2^ to 10^−8 ^M over 10 days and IC_50_ values were calculated (Fig. 3a, c). The IC_50_ of DRB was higher in MOSJ-Dkk-1 cells at all time points with the greatest reduction in IC_50_ occurring at day 4 for both cell lines (Fig. 3a). The day-4 timepoint corresponded to the initiation of the rapid logarithmic phase of growth (Fig. 3b, plot) and the difference between the IC_50_ in MOSJ-Dkk-1 cells versus MOSJ-pLenti cells was greatest at this point (Fig. 3b, bars). It was therefore hypothesised that Dkk-1 blockade by DkkMo could increase the effectiveness of DRB during periods of rapid mitosis. This was tested by exposure of cultures of proliferating MOSJ-Dkk-1 cells to combinations of DRB (at IC_50(day4)_) and DkkMo (2.5 and 5.0 μM) for 2, 4, 6 and 9 days. DRB alone killed approximately half of the cells, but in the presence of 5.0 μM DkkMo, this rate increased to approximately 100% at all time points (Fig. 3d). DkkMo at 5.0 μM also possessed an intrinsic capacity to reduce the accumulation of MOSJ-Dkk-1 cells to a degree comparable to DRB alone. DkkMo at a dose of 2.5 μM exhibited similar activity, but this was confined to day-4 cultures. These data demonstrated that DkkMo could inhibit the expansion of MOSJ-Dkk-1 cells and significantly sensitised the cells to DRB.

#### DkkMo inhibits MOSJ-Dkk-1 tumour expansion and induces necrosis in vivo

**Fig. 4:**
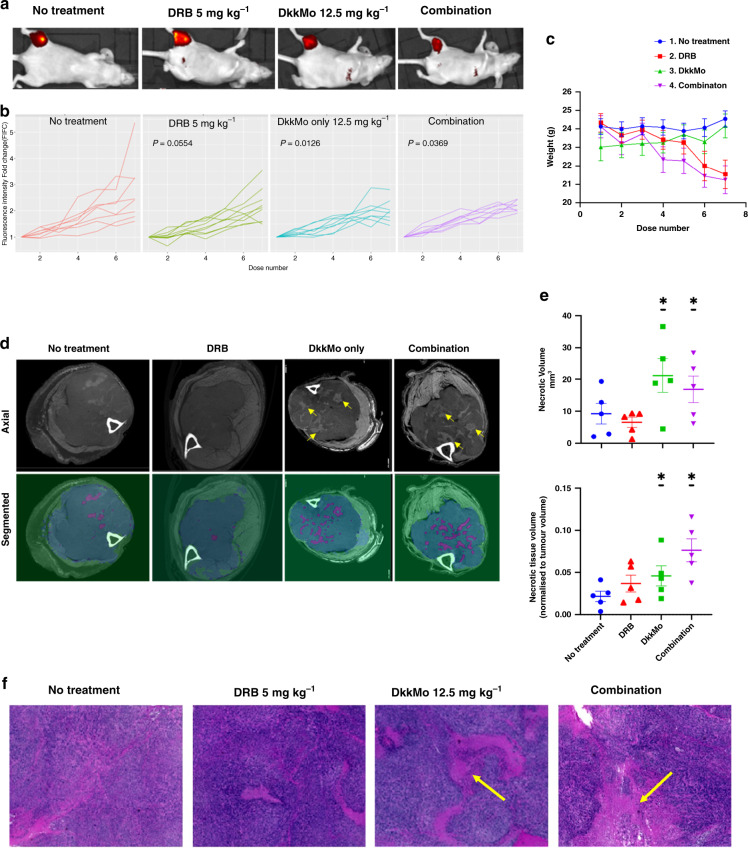
DkkMo administration reduces the rate of expansion of MOSJ-Dkk-1 tumours and causes the generation of necrotic foci. **a** Fluorescent imaging of nude mice harbouring orthotopic tumours of dsRed-labelled MOSJ-Dkk-1 cells. **b** Plots of tumour expansion as a function of fold-change fluorescence intensity. Each line represents a single tumour (*n* = 8–9). *P*-values calculated by mixed-model regression are compared to the no treatment condition. **c** Plots of animal weight throughout the experiment (*n* = 8–9). **d** Axial images (above) of tumours at proximal/distal midpoint indicating larger numbers of radio-opaque foci in the DkkMo-treated conditions (examples, arrowed). Segmentation of the images (below) based on automated recognition by CNNA. Bone (white), soft tissue (green), tumour (blue) and foci (cyan) are indicated. **e** Volumetric measurements of radio-opaque foci using the CNNA (*n* = 5). Volumes per tumour (above) and the volume of necrotic foci normalised to tumour volume (below). The volumes per tumour (above) were analysed by a generalised linear model account for tumour size. The volume of necrotic foci normalised to tumour volume (below) analysed by beta regression (*Padj < 0.05). **f** H- and E-stained sections of tumours indicate that radio-opaque structures are acellular foci of necrotic tissue (examples arrowed).

Fluorescently labelled (dsRed) MOSJ-Dkk-1 OS were initiated in hindlimbs of immune-deficient nude mice followed by administration of DRB (5 mg kg^−1^), DkkMo (12.5 mg kg^−1^), or combination, by intraperitoneal injection every 48 h. The DRB dose was the highest that could be administered without significantly affecting survival (Supplementary Fig. S1A). Since scrMo had no effect on MOSJ-Dkk-1 cells in vitro, and the systemic effects of the backbone morpholino are negligible [33, 34], control (no treatment) mice received saline only. Tumour expansion was followed by measurement of fluorescent intensity normalised to time = 0 (Fig. 4a, b). In each case, the slopes (representing growth rate) were compared using a mixed model for repeated measures (MMRM) approach (Fig. 4b and Supplementary Fig. S1B). Combination therapy and DkkMo alone reduced the rate of tumour expansion as compared to the untreated control group but co-administration of DRB did not appear to improve the effect of DkkMo. The lack of observed synergy or additive contribution between DRB and DkkMo could result from DRB stimulating Aldh1a1 through an alternate pathway, but DRB alone did not increase Aldh1a1 transcription even though endogenous murine Dkk-1 was slightly upregulated (Supplementary Fig. S1C–E). It is also noteworthy that DkkMo administration did not affect the weight of the mice, whereas DRB toxicity resulted in significant weight loss (Fig. 4c). After 2 weeks of treatment, hindlimbs harbouring tumours were dissected and stained with Lugol’s iodine contrast agent. Upon inspection of the scans, radiodense patches were observed in the tumour masses that were more prevalent in tumours that received DkkMo and combination treatment (Fig. 4d, arrowed). A convolutional neural network algorithm (CNNA) was trained to segment the tumour and the radiodense patches on the axial reconstructions (Fig. 4d and Supplementary Fig. S3). When the CNNA was employed to measure the volume of radiodense structures in all tumour specimens and normalise this to total tumour volume, there was a significant increase in tumours that were treated with DkkMo or combination (Fig. 4e and Supplementary Fig. S3). MOSJ-Dkk-1 tumours consist of a pleomorphic mass of tightly packed fibroblastoid cells that resemble high-grade undifferentiated OS tumours associated with poor prognosis (Supplementary Fig. S4A–C). In contrast, histology of the radiodense structures indicated acellular foci (Fig. 4f and Supplementary Fig. S4D–G, asterisks) that had become infiltrated with a collagenous matrix which stained a characteristic blue with Masson’s trichrome stain (Supplementary Fig. S5). Cells adjacent to the acellular foci often exhibited signs of pyknosis and nuclear fragmentation characteristic of necrosis (Supplementary Fig. S5C). Collectively, the data indicated that the radio-opaque structures were collagenized necrotic foci and that DkkMo not only slowed tumour expansion but also had the capacity to induce tumour necrosis.

#### DkkMo reduces bone destruction in vivo

**Fig. 5:**
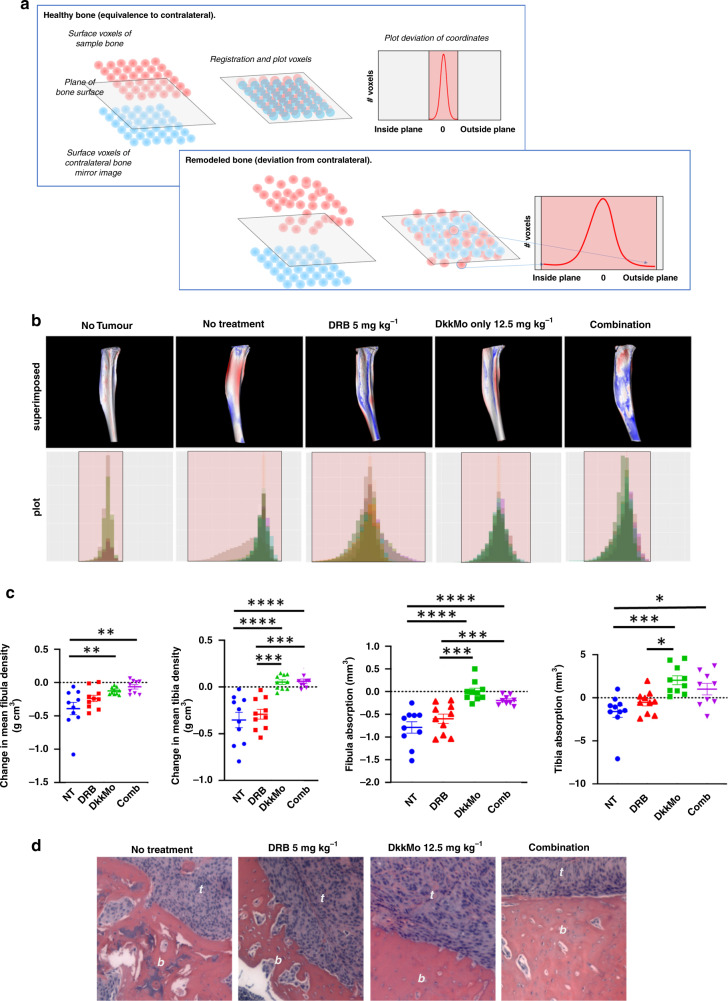
DkkMo administration reduces the rate of bone destruction by MOSJ-Dkk-1 tumours. **a** Diagrammatic explanation of the principle of the topological analysis employed to measure bone remodelling. **b** Topological comparisons of tumour-bearing tibiae with unaffected contralateral tibiae demonstrate DkkMo treatment preserves the original shape of the bone tissue. Rendered scans (above) indicate where surface of the tumour-laden tibia is inside (red) or outside (blue) the plane of the surface of the contralateral tibia. Distribution plots (below) with individual samples depicted in shades of brown, grey or green with the maximum width of the distribution depicted in pink. Healthy bones result in narrow distributions whereas damaged bones generate asymmetrical and broad distributions. *P*-values represent statistical comparisons of plots versus no treatment. **c** Bone density for tumour-laden tibiae and femora (leftmost two panels) plotted as deviation from the contralateral measurements. Bone absorption for tumour-laden tibiae and femora (rightmost two panels) plotted as deviation from the contralateral measurements. *P*-values calculated by ANOVA with Tukey multiple comparison tests (*n* = 8). *P*adj < 0.05 = *, *P*adj < 0.01 = **, *P*adj < 0.005 = ***, *P*adj < 0.001 = ****. **d** H- and E-stained sections of tumour bone interfaces indicate reduced bone erosion in DkkMo-treated tumours. The bone (*b*) and tumour (*t*) are labelled in each case.

Dkk-1 has the capacity to prevent the bone repair in OLs [10, 12] and MOSJ-Dkk-1 cells generate aggressive and highly osteolytic tumours in mice [15]. To examine whether DkkMo treatment could reduce the osteolytic effects of MOSJ-Dkk-1 cells, bones in the tumour-bearing hindlimbs of mice were scanned by μCT. Qualitative inspection of tibias and fibulas indicated that MOSJ-Dkk-1 tumours caused significant destruction. Superimposition of scanned images of malformed bones onto unaffected contralateral control scans highlighted where, and to what extent, the surface topology of the bone had deviated from the wild-type form. By measuring these deviations, a profile could be generated illustrating the frequency of voxels that had deviated outwards or inwards from the plane of the healthy bone surface. A healthy bone measurement generates a narrow profile, indicating that few voxels deviated from the plane of the contralateral control bone, and if so, by a small degree (Fig. 5a, above). A deformed bone would generate a broader distribution, indicating a surface topology consisting of many voxels above and below the plane of the surface of the contralateral control bone (Fig. 5a, below). When applied to tibial specimens, healthy bones generated the expected narrow profile whereas untreated MOSJ-Dkk-1 bearing tibias demonstrated a high degree of deformation, generating broad profiles with the same true for DRB-treated specimens (Fig. 5b). In contrast, tibias from tumour-bearing mice that received DkkMo or combination therapy generated narrower profiles, indicating less deformation (Fig. 5b). Statistical analysis of the plots demonstrated that when compared to the no treatment group, the DkkMo plots differed in terms of *x* axis positioning (*P*adj = 0.018 with DkkMo, distributions significantly closer to zero) and breadth of the distribution (*P*adj = 0.05 with DkkMo distributions significantly narrower), indicating that DkkMo reduced bone deformation. The same trend was evident for the combination group, but p-values were larger (*P*adj = 0.079 for *x* axis, *P*adj = 0.192 for distribution), suggesting that DRB might hinder the osteoprotective action of DkkMo. MicroCT measurement of the tibias and fibulas further confirmed that DkkMo reduced a tumour-associated drop in bone mineral density (Fig. 5c, left) and absorptive destruction of the bone tissue (Fig. 5c, right). Osteolytic activity of MOSJ-Dkk-1 cells was widespread in untreated and DRB-treated specimens, but this was less apparent in specimens that received DkkMo or combination (Fig. 5d).

#### DkkMo modulates proliferative, migratory, survival and immunological processes in vivo

**Fig. 6:**
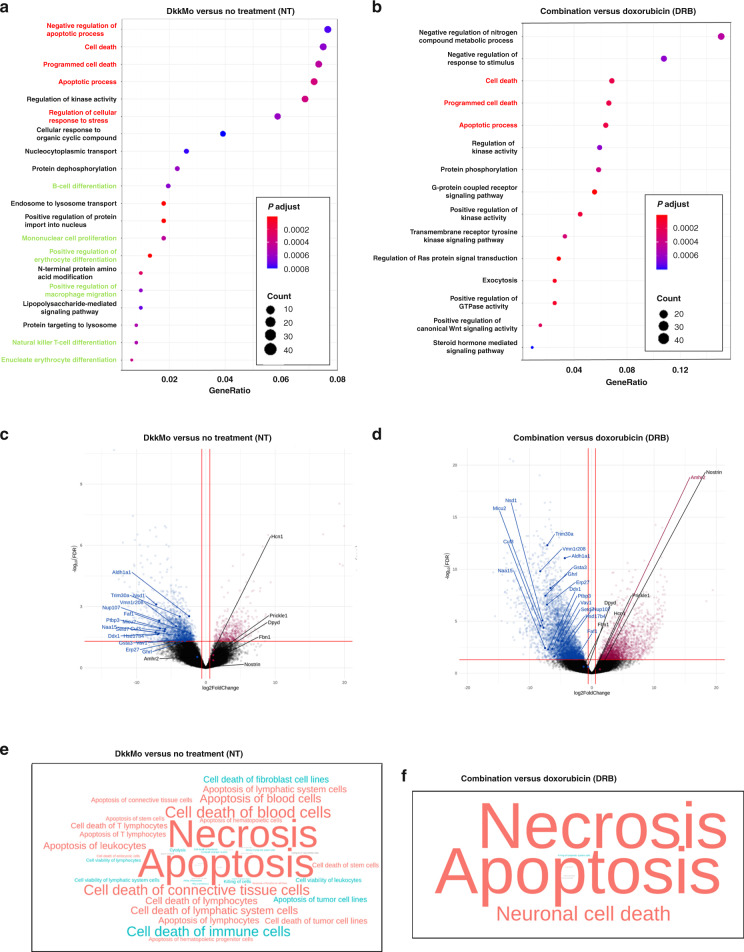
DkkMo administration blocks stress response pathways in orthotopically implanted MOSJ-Dkk-1 tumours. **a** Gene ontology term enrichment analysis of RNA sequencing data with the comparison between DkkMo and no treatment (left). **b** As panel **a**, but with the comparison of DRB and combination treatment (right) (*n* = 4). Red font refers to groups related to cell death and survival and green font refers to immune-related genes. **c** Volcano plot of DE genes after comparison of the DkkMo with the no treatment group. Sequences that represent both DE and hub genes are labelled. **d** As panel **c**, but the plot of DE genes after comparison of the DRB group with the combination group. **e** Word cloud summarising IPA results using DE gene lists from the comparison of the DkkMo with the no treatment group. Colour and font coding as in Fig. 2. **f** As panel **e**, but with DE genes after comparison of the DRB group with the combination group.

To gain insight into the anti-tumorigenic mechanism of action of DkkMo, tumours were excised and subjected to HTS. Comparison between untreated (NT) and DkkMo specimens (*n* = 4) and comparison between DRB-treated and combination specimens (*n* = 4) were performed. After processing, lists of DE transcripts were generated (Supplemental Table 2). When the lists were subjected to gene ontology term enrichment analysis, clusters representing regulation of cell stress, death and apoptosis were highlighted in both comparisons, further supporting a role for Dkk-1 in the regulation of stress response and survival pathways, and that DkkMo has the capacity to blunt such activities (Fig. 6a, b). Unexpectedly, gene ontology term enrichment analysis of the DkkMo versus NT groups highlighted several gene ontologies related to differentiation of T cells, macrophages and erythrocytes (Fig. 6a). Co-expressed, functionally related gene modules and hub genes were also identified (Supplementary Fig. S6) and plotted on volcano plots to visualize potential overlap between lists of DE genes (Fig. 6c, d). As with the in vitro analyses, Aldh1a1 and Hsd17b4 were represented in both comparisons as hub and DE genes. Also of note were histone methyltransferases Setd7 and Nsd1, both implicated as mediators of oncogenesis [35, 36]. IPA indicated that DkkMo treatment promoted upregulation of genes involved in necrosis and apoptosis in the case of both comparisons (Fig. 6e, f). In the case of the NT versus DkkMo comparison, IPA also highlighted several gene groups associated with the death of blood cells and connective tissue cells. Collectively, the IPA data suggest that DkkMo may inhibit tumorigenesis by depleting cells in the microenvironment as well as by modulating survival capacity.

To examine whether DkkMo could perturb genes involved with cell motility and migration, differentially expressed genes in the NT versus DkkMo group and in the DRB versus combination groups were plotted against selected GO terms related to cell migration, attachment and metastasis. DkkMo perturbed gene expression over several of these GO terms, with particular emphasis on actin reorganisation (Supplementary Fig. S7). Furthermore, in both comparisons, Actin Related Protein 2/3 Complex Subunit 2 (Arpc2), a major component of the actin polymerisation complex, and potent mediator of cell migration and metastasis [37, 38], was the most downregulated transcript. To further examine whether DkkMo perturbed the capacity for cell migration, scratch assays were performed on monolayers of MOSJ-Dkk-1 cells. In the presence of scrMo or with no treatment, 0.5 mm-wide scratches closed in 60–80 h through the combined process of proliferation and migration but scratches treated with 25 μM DkkMo failed to close in over 80 h (Supplementary Fig. S8A, B). Counts of cells that had migrated into the scratch zone after 24 h were also significantly lower in DkkMo-treated monolayers (Supplementary Fig. S8C).

Aldh1a1 is a marker for a cancer stem cell or tumour initiator phenotype [16, 39] and it is possible that elevated Aldh1a1 expression is a sign that Dkk-1 has the capacity to induce this in all or a subset of MOSJ-Dkk-1 cells. We, therefore, analysed transcriptomic datasets for differential expression of 30 known OS cell markers, testing the hypothesis that if this were the case, additional stem cell markers may be downregulated in response to DkkMo (Supplementary Table S3). In most cases, but with the exception of Aldh1a1, there was no significant change in transcription after treatment with DkkMo. Interestingly, cancer stem cell marker Sox2 exhibited a robust reduction in transcription after DkkMo treatment under in vitro conditions. These data provide modest evidence of the involvement of Dkk-1 in the initiation of a cancer stem cell phenotype, but Dkk-1, Sox2 and Aldh1a1 have been reported to exhibit coordinated expression to maintain a stem cell phenotype in carcinoma cells [40].

## Discussion

The first association between Dkk-1 and osteolytic malignant bone disease was demonstrated in multiple myeloma [12] then for OS [10] and metastases of breast, prostate and lung cancers [13, 14, 41–43]. Dkk-1 was subsequently shown to block the anabolic axis of bone turnover, suggesting that inhibition of Dkk-1 may inhibit the development of OLs. Initial strategies for the blockade of Dkk-1 began with antibodies, and these showed promise in myeloma models [20] and in humans [44], but antibodies must be given at large doses given that Dkk-1 levels in humans can reach hundreds of ng mL^−1^ [10, 12]. To circumvent limitations of antibodies, this study employed a vivo morpholino directed against human Dkk-1 mRNA. Vivo morpholinos consist of an oligonucleotide mimetic attached to a dendrimeric moiety that facilitates cellular internalisation. While originally utilised for simple organisms [45], vivo morpholinos have been increasingly employed for gene knockdown in mammalian species such as rodents, dogs and even humans [34]. Indeed, an exon-skipping vivo morpholino was first successfully tested as a treatment for Duchenne Muscular Dystrophy in 2009 [46] and shortly thereafter exon-skipping morpholinos were approved in the USA and Japan. DkkMo is directed to the 5’ coding region of human Dkk-1. When MOSJ-Dkk-1 cells were exposed to DkkMo, Dkk-1 secretion was dose-dependently inhibited and the rate of accumulation of cells in culture was reduced and when confluent MOSJ-Dkk-1 cells were treated with DkkMo, cell death occurred, phenocopying the parental and control cell lines. These data indicate that Dkk-1 plays a role in the survival of MOSJ-Dkk-1 cells under conditions of rapid proliferation and nutritional stress. In our previous study [15] and herein, Dkk-1 was demonstrated to elevate expression of Aldh1a1 by diverting Wnt signalling from cWnt to a ncWnt pathway involving RhoA and JNK. Activated JNK triggers Jun to bind to the Aldh1a1 promoter, upregulating its expression. Aldh1a1 then acts to enhance resistance to nutritional and chemotherapeutic stressors in part by neutralisation of ROS [15]. The role of the ALDH family of enzymes in driving the survival, progression and metastasis of tumours is well documented [22, 26–29, 31, 47], but the means by which it is upregulated in OS is controversial, especially with respect to cWnt signalling. For example, Martins-Neves et al. [48] reported that chemotherapeutics cause β-catenin stabilisation and concomitant transcription of TCF/LEF promoters, including expression of Aldh1a1. The study also noted that the chemotherapeutics further triggered the expression of Dkk-1, potentially through a negative-feedback mechanism driven by cWnt responsive promoters in the Dkk-1 gene. In spite of the apparent contradiction between the findings of Martins-Nevens et al. positing β-catenin mediated upregulation of Aldh1a1, and the ncWnt/JNK pathway described in our previous work, these pathways can theoretically contribute in parallel to the upregulation of Aldh1a1. Recently, the cytoskeleton-associated protein 4 (CKAP4) receptor has been shown to serve as a receptor for Dkk-1, signalling via phosphatidylinositol-3-kinase to activate Akt [49]. Akt has the capacity to inhibit glycogen synthease kinase-3-β, stabilising β-catenin, potentiating upregulation of cWnt target genes, including Aldh1a1 [50]. This hypothesis does not exclude a parallel function for ncWnt-stimulated JNK in the upregulation of Aldh1a1. Regardless of the mechanism, the regulatory relationship between Dkk-1 and Aldh1a1 expression is demonstrated by the observation that DkkMo reduces Aldh1a1 transcription and Aldh1a1 is identified as a DE and hub gene that is downregulated in the presence of DkkMo.

From the in vitro HTS studies, two hub and DE genes were identified with the potential to contribute to tumour progression. Hsd17b4, encoding 17β-hydroxysteroid dehydrogenase/D-3-hydroxy acyl-coA dehydrogenase, is downregulated when treated with DkkMo. Hsd17b4 is responsible for deactivation of oestrogens and androgens as well as oxidation of fatty acids and other complex substrates [30]. It is conceivable that it plays a role in the oxidation of long-chain or complex acids that result from the processing of reactive aldehydes by Aldh1a1. DkkMo also downregulated S1pr2, encoding sphingosine-1-phosphate-receptor-2, regulator of osteoblast chemotaxis and inverse marker of osteoblast differentiation [51].

Since DkkMo has the capacity to sensitise MOSJ-Dkk-1 cells to stress, DkkMo may synergise with chemotherapeutics that induce oxidative damage. DRB is frequently employed as an adjuvant treatment for OS, and is known to cause oxidative damage through induction of ROS [52]. In the presence of DRB, MOSJ-Dkk-1 cells expanded more rapidly than MOSJ-pLenti cells and the difference in IC_50_ values between MOSJ-Dkk-1 cells and MOSJ-pLenti cells was also greatest when the rates of proliferation were highest. These results suggested that DRB is at its most toxic during proliferation and that Dkk-1 provides most protection at this time, concurring with reports that chromatin is susceptible to DRB-induced damage during mitosis [52] and Dkk-1 levels are elevated in cycling OS and mesenchymal cells [53, 54].

Given the role of cWnt in the stimulation of proliferation in many systems and cancers [55], a pro-tumorigenic role for Dkk-1 is surprising but quite feasible given the multiple roles for cWnt, the countless variants of Wnts and various non-canonical pathways. Unlike those Wnt inhibitors that directly sequester Wnt ligands such as soluble frizzled-related protein (sFRP) and Wnt inhibitory factor (WIF), Dkk-1 is unique in its capacity to act independently of Wnt through LRP6, JNK, CamKII [56] and through its ability to directly engage CKAP4 and trigger PI3K signalling [49]. While Dkk-1 can serve as a tumour suppressor in some cases by inhibition of cWnt [56] the complications of Dkk-1 signalling make its specific effect on tumour physiology unpredictable and there are several reported examples of Dkk-1 serving a pro-tumorigenic role through enhancement of growth and/or survival [15, 54, 57, 58], migratory [59], and metastatic [21, 54] characteristics. Of note, Colla et al. [60] demonstrated upregulation of Dkk-1 by human myeloma cells when challenged by ROS generating chemotherapeutics resulting in upregulation of JNK. While Dkk-1 cannot be formally regarded as a proto-oncogene, the data herein suggest that Dkk-1 certainly appears to serve as a survival factor for tumours.

Orthotopically implanted MOSJ-Dkk-1 cells rapidly generate tumours in nude mice. When DRB was administered at a high dose, tumour expansion was not significantly affected, whereas DkkMo administered as a single agent significantly blunted tumour growth. Given that synergy was observed between DRB and DkkMo in vitro, it is surprising that it was not observed in vivo. One explanation is that DRB triggers expression of endogenous Dkk-1 from MOSJ cells and this in turn competes with DkkMo. While DRB did upregulate endogenous Dkk-1 transcription by about 30%, the expression of murine Dkk-1 is negligible compared to the human form expressed by MOSJ-Dkk-1 cells [15]. The in vitro assays, however, do not take into account potential sources of Dkk-1, Aldh1a1 or other stress response agents derived from the tumour stroma which may not be targeted by DkkMo.

MOSJ-Dkk-1 cells form highly osteolytic tumours in nude mice accompanied by significant bone deformation. We employed μCT scans to perform quantitative topological comparisons between the contralateral and tumour-bearing tibiae and found that untreated tumours exhibited substantial bone deformation that was exacerbated by DRB. When DkkMo was administered, the bone exhibited a healthier topological profile, even in the presence of DRB, indicating that DkkMo may act to protect bone from detrimental effects of treatments as well as the tumour. While immunological blockade of Dkk-1 preserves bone in experimental models [20] and multiple myeloma patients [44], this is the first time Dkk-1 blockade has been shown to preserve topology as well prevent bone loss.

Tumours were subjected to HTS to gain mechanistic insights into the effect of DkkMo. The dsRed label was utilised to exclude as much extraneous tissue as possible, but the HTS data originating from in vivo specimens contrasted significantly with data from cultured cells. Differences in gene expression between the in vivo and in vitro HTS datasets are likely to be attributable to the contribution of host tissue such as blood, tumour-associated stroma and immune cell contributions secondary to responses to the host. Nevertheless as observed in vitro, DkkMo upregulated cell death-related gene ontologies, with Aldh1a1 and Hsd17b4 both identified as downregulated transcripts and as hub genes. The in vitro and in vivo detection of Aldh1a1 and Hsd17b4 as downregulated sequences with DkkMo treatment, and their potentially interrelated roles in the metabolism of organic substrates, suggests a novel role for Hsd17b4 as a mediator of drug resistance. Unexpectedly, there was representation from immune and hematopoietic GO terms with DkkMo treatment, implying that Dkk-1 might also regulate immune processes. These data should be regarded with some caution given the T-cell-depleted status of nude mice, but Dkk-1 has been reported to stimulate infiltration of macrophages and neutrophils [61, 62] and stimulate immunosuppressive myeloid suppressor cells to facilitate evasion from immune surveillance [63], and these cell-types are present in nude mice. Furthermore, the IPA data highlighted upregulation of sequences associated with the death of immune, lymphatic, connective tissue upon treatment with DkkMo, suggesting that Dkk-1 blockade might contribute to the depletion of the tumour stroma.

MOSJ-Dkk-1 cells are an excellent model to study primary OS expansion and bone destruction, but little is known about their ability to metastasize. Attempts to detect MOSJ-Dkk-1 cells in the lungs of mice by PCR were unsuccessful, but DkkMo perturbed expression of several genes involved in cell attachment, migration and metastasis of tumours. This included downregulation of Arpc2, a regulator of actin polymerisation and mediator of tumour migration in several tissues [37, 38, 64]. DkkMo also perturbed migration on monolayers suggesting that cytoskeletal mediators of cell motility had been affected. While these data not provide definitive proof that DkkMo inhibits metastasis, it should be noted that immunoblockade of Dkk-1 reduced lung metastasis in a patient-derived xenograft model of OS metastasis to lung [21] and Dkk-1 has been shown to promote migration and invasive growth [59, 65, 66].

Small interfering RNA has been employed to block Dkk-1 and exhibit neuroprotection in a model of intracerebral haemorrhage [67], inhibit inflammation in a model of rheumatoid arthritis [68], and in a model of hormone deficiency-induced bone loss [69], but to the best of our knowledge, this is the first demonstration of the use of a Dkk-1 targeting vivo morpholino to treat experimental malignant bone disease. Using a murine model of a Dkk-1 expressing OS, we demonstrated that DkkMo reduces tumour progression through reduction of cell proliferation, perturbation of survival mechanisms, inhibition of bone destruction, and through depletion of the tumour stroma. Given that morpholinos have a robust half-life in vivo and are well tolerated, DkkMo represents a promising approach for the improvement of OS treatment in humans.

## Supplementary information

Supplemental tables

Figure S1

Figure S2

Figure S3

Figure S4

Figure S5

Figure S6

Figure S7

Figure S8

Supplemental materials

## Data Availability

Until the archive is completed, all raw data are available upon request. All raw data will be provided in a Raw Data File uploaded to FigShare.com [10.6084/m9.figshare.18551354]. Sequencing data are available via the Gene Expression Omnibus [GSE191143].
