# Supplementary figures and images for: Morpholino-driven blockade of Dkk-1 in osteosarcoma inhibits bone damage and tumour expansion by multiple mechanisms
Source: Br J Cancer. 2022 Mar 11;127(1):43–55. doi: 10.1038/s41416-022-01764-z (PMC9276700; doi:10.1038/s41416-022-01764-z)

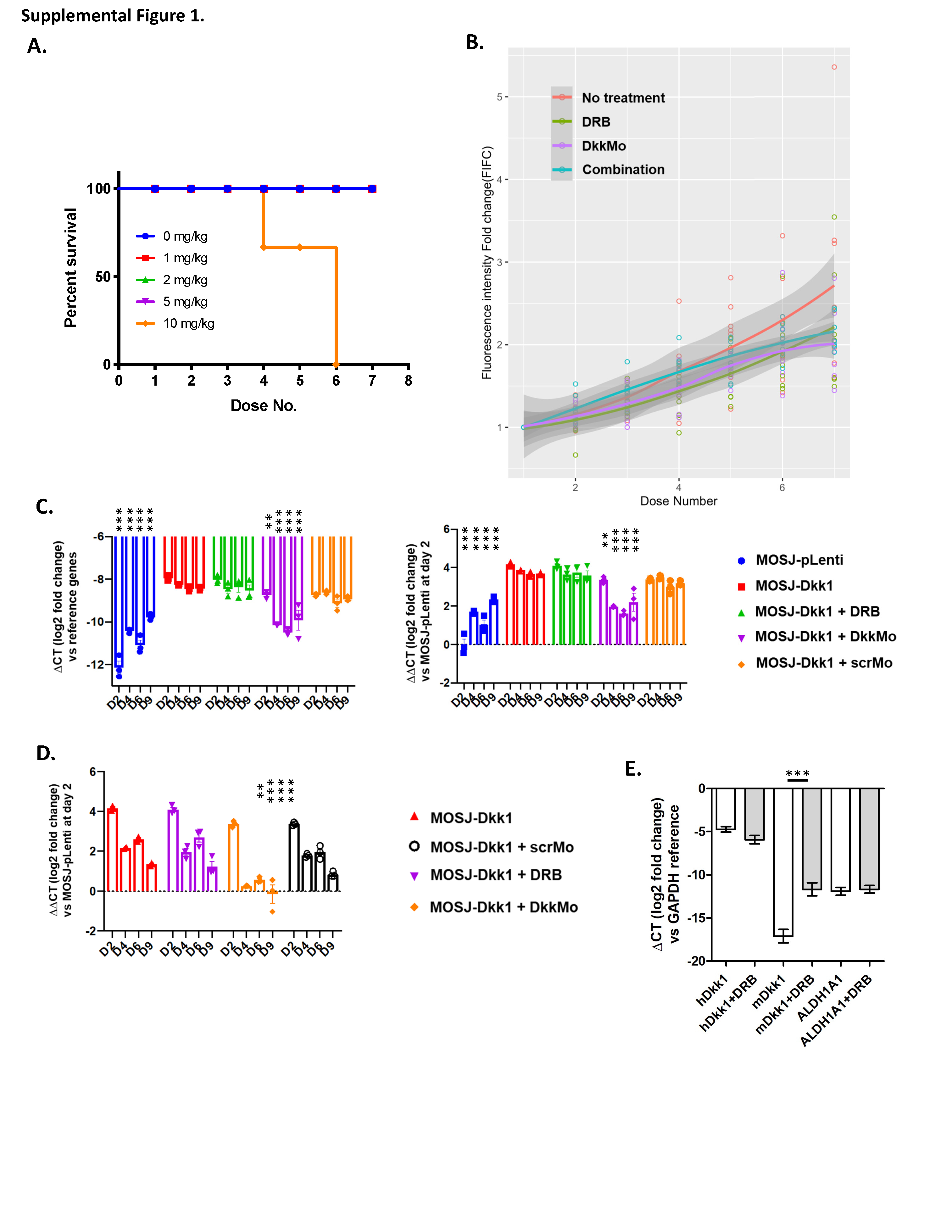

Supplement: Supplementary file 2 — Figure S1 [file 41416_2022_1764_MOESM2_ESM.tif]

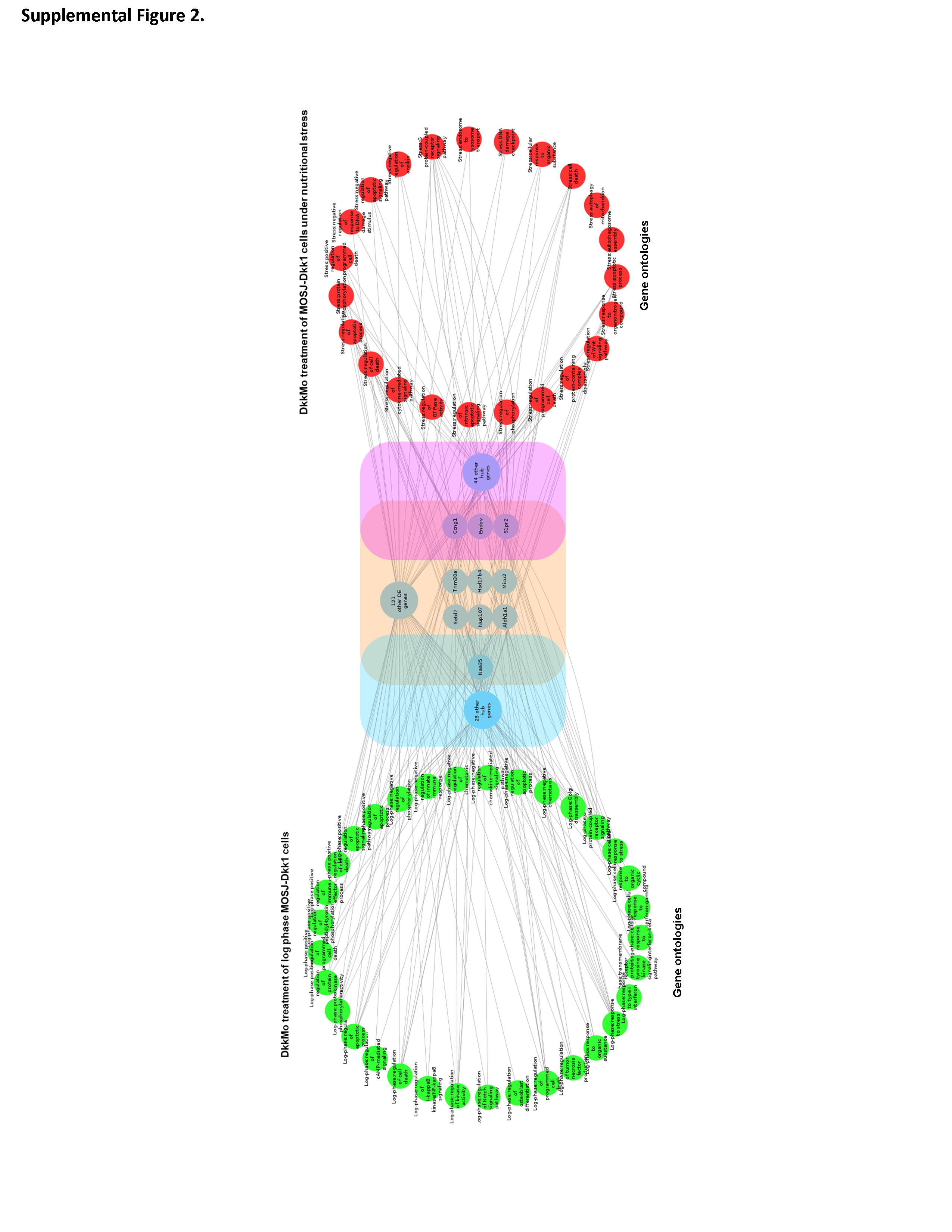

Supplement: Supplementary file 3 — Figure S2 [file 41416_2022_1764_MOESM3_ESM.tif]

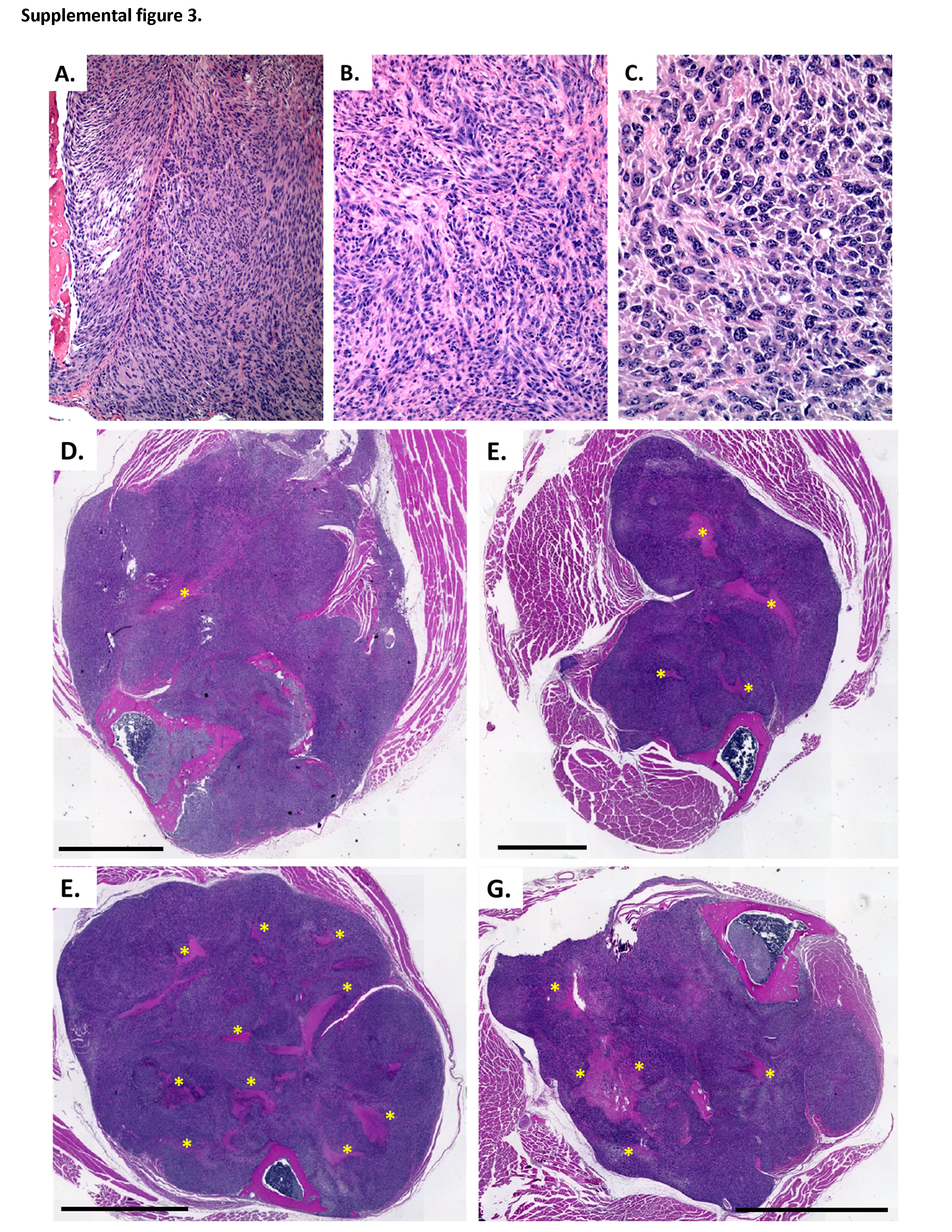

Supplement: Supplementary file 4 — Figure S3 [file 41416_2022_1764_MOESM4_ESM.tif]

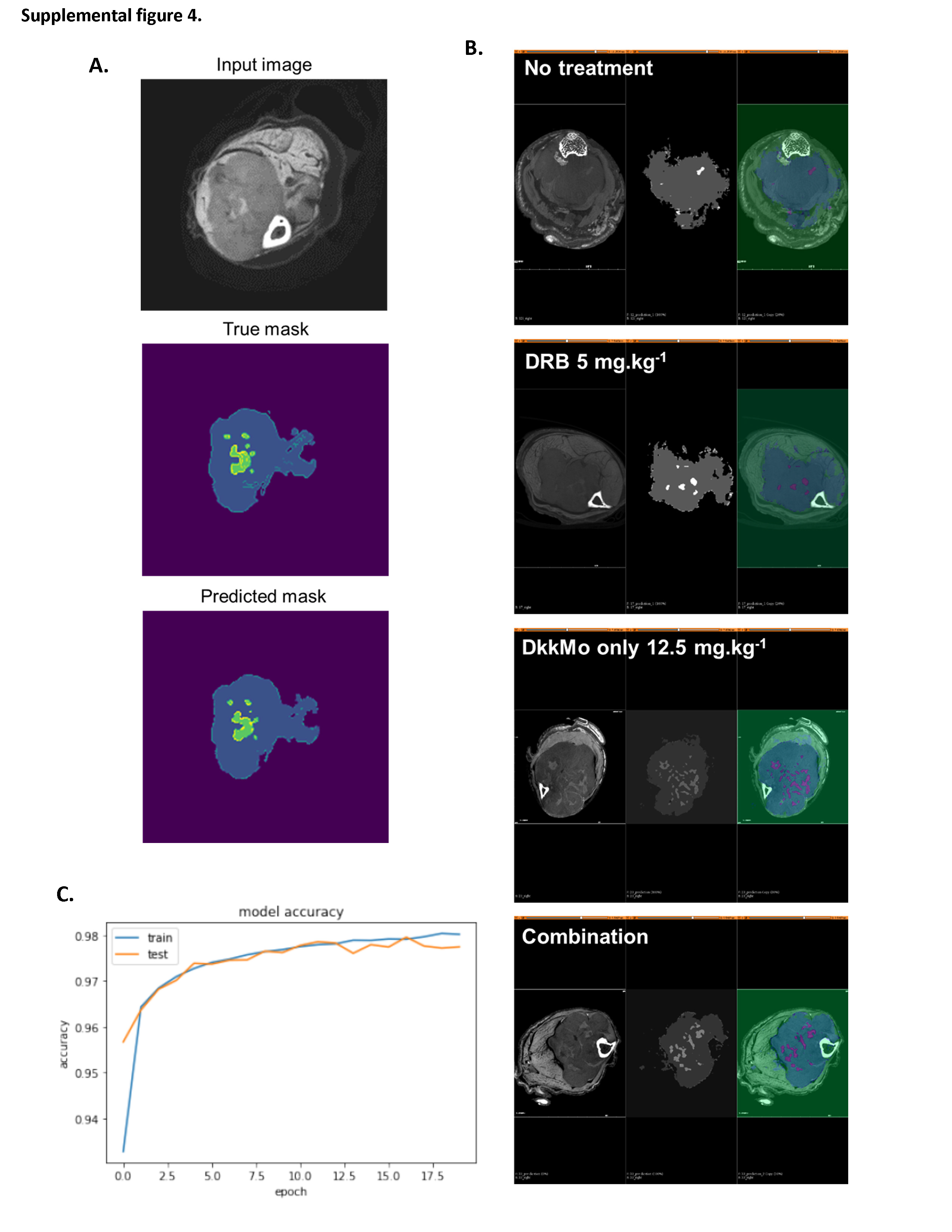

Supplement: Supplementary file 5 — Figure S4 [file 41416_2022_1764_MOESM5_ESM.tif]

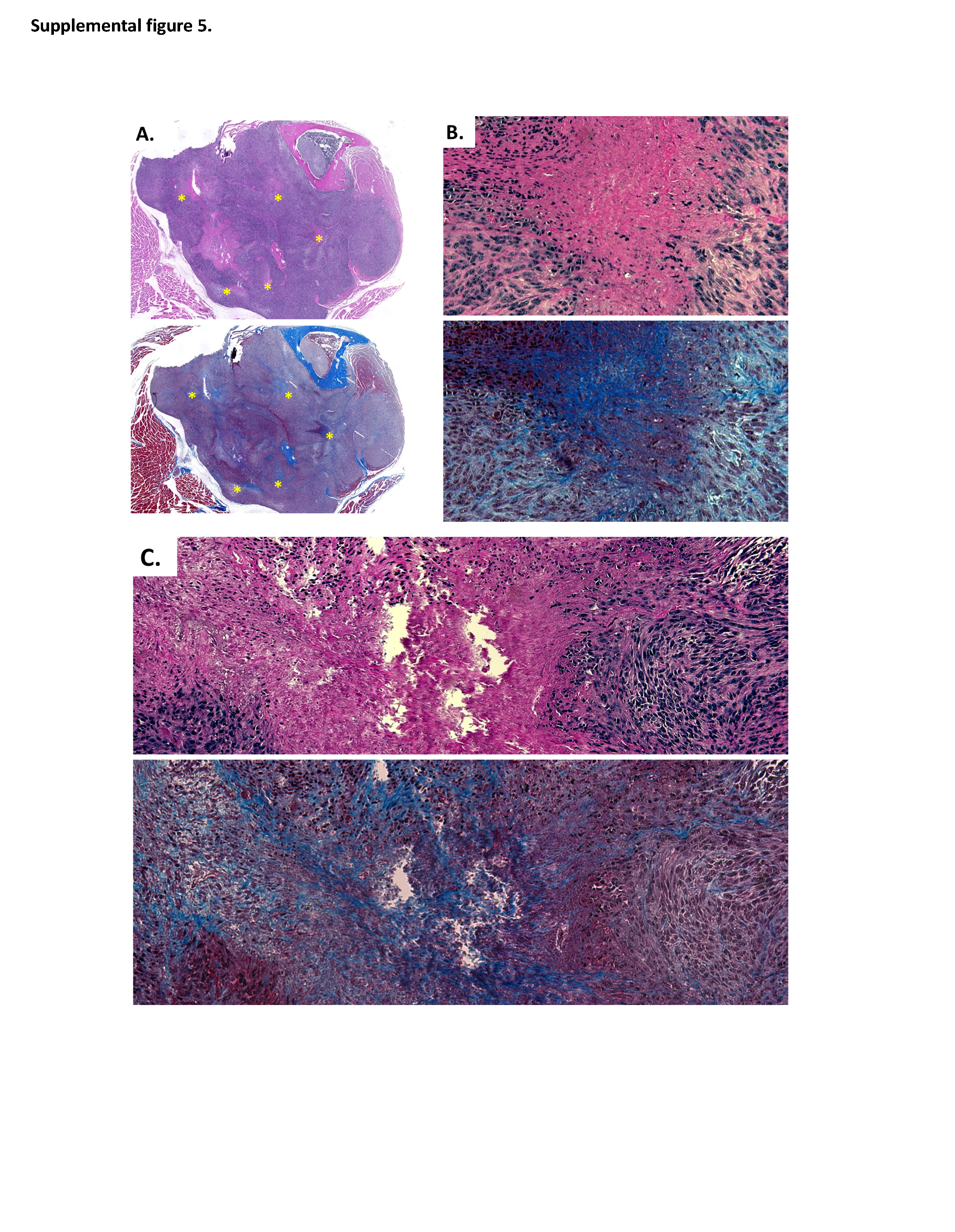

Supplement: Supplementary file 6 — Figure S5 [file 41416_2022_1764_MOESM6_ESM.tif]

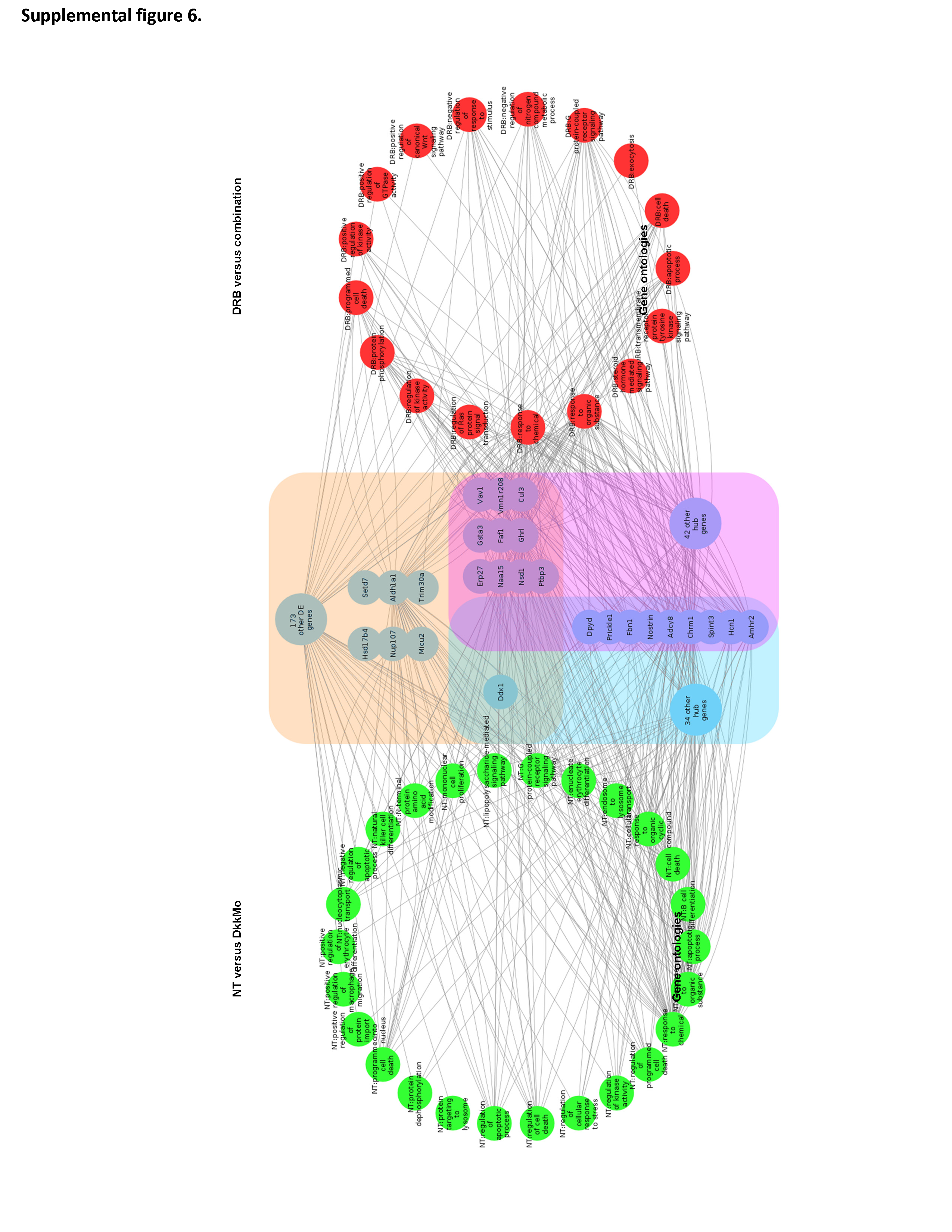

Supplement: Supplementary file 7 — Figure S6 [file 41416_2022_1764_MOESM7_ESM.tif]

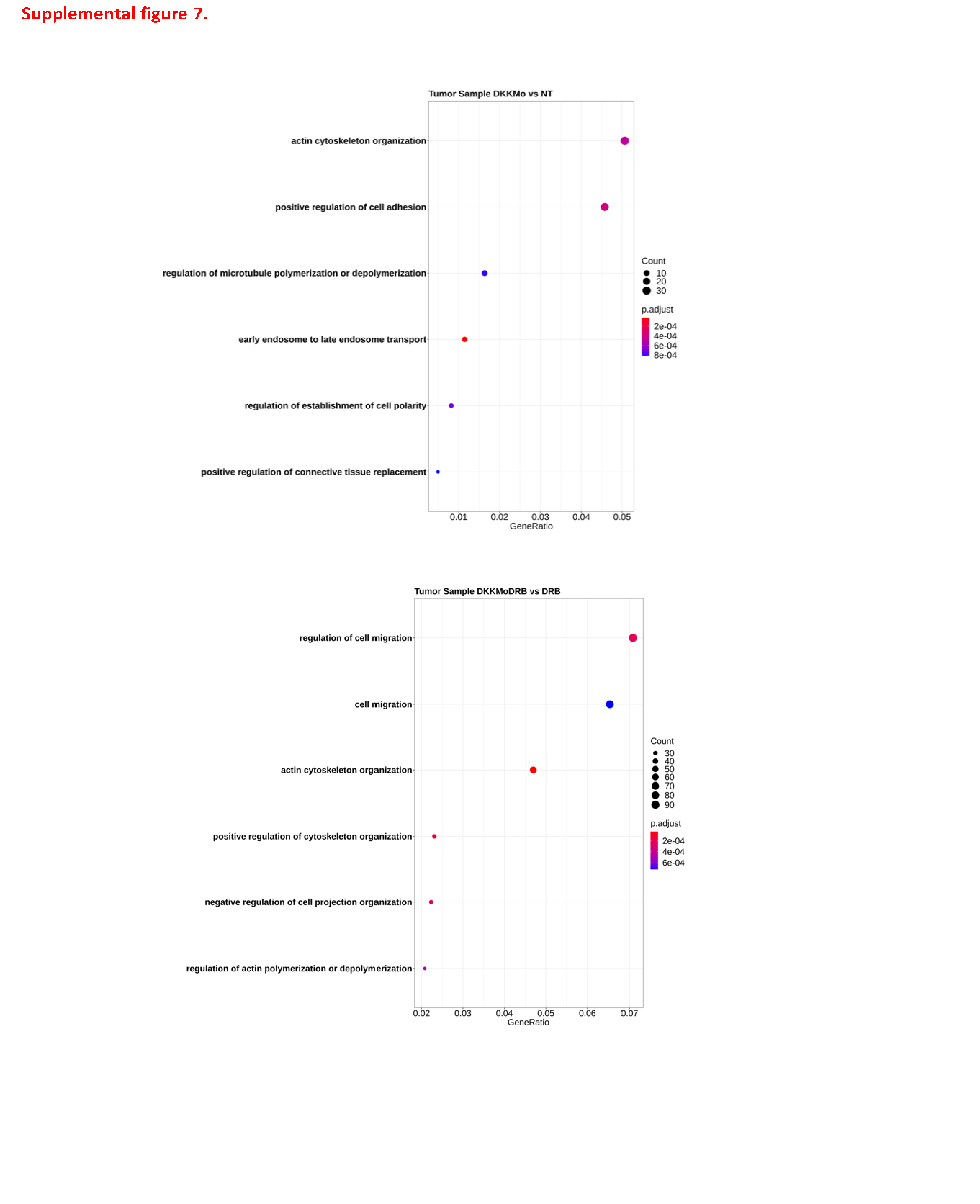

Supplement: Supplementary file 8 — Figure S7 [file 41416_2022_1764_MOESM8_ESM.tif]

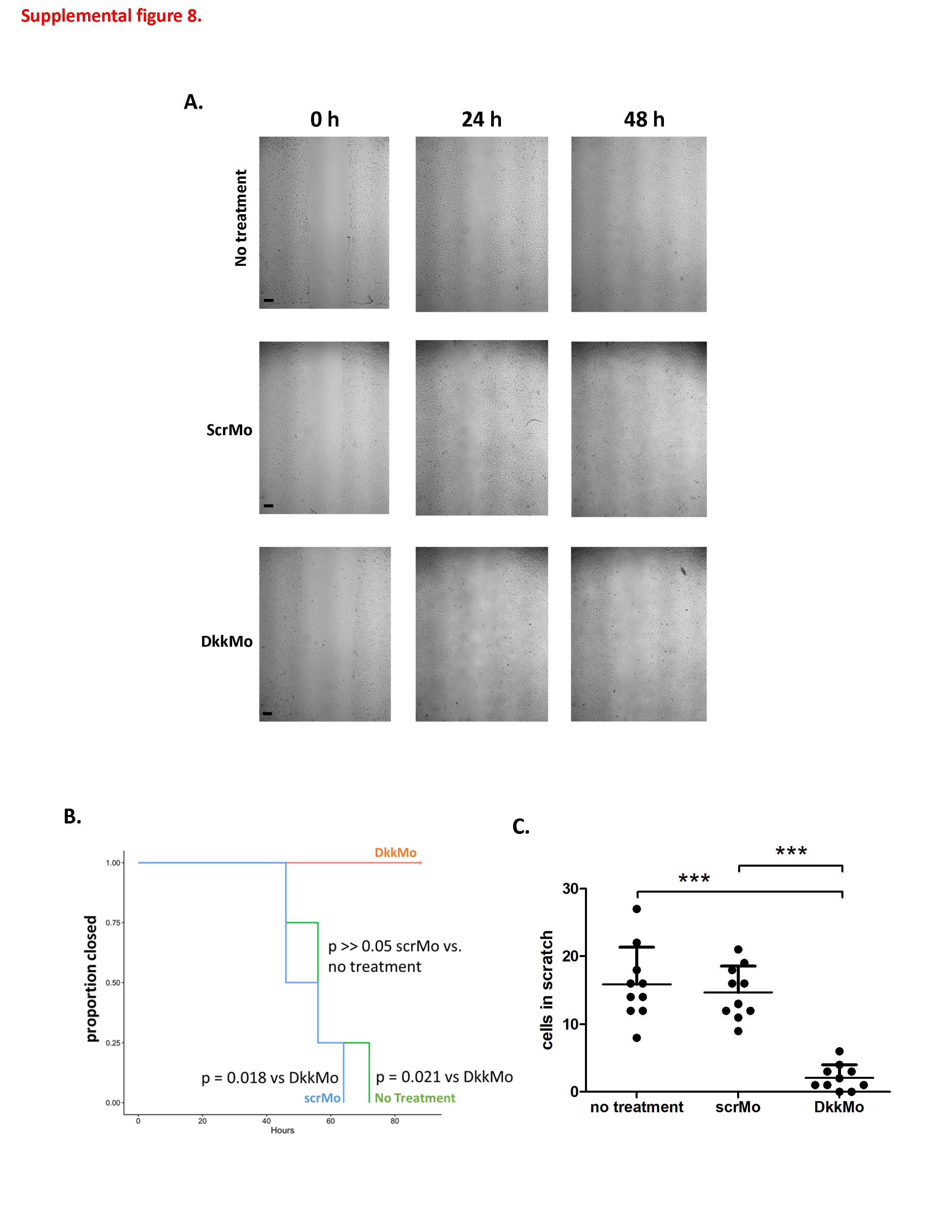

Supplement: Supplementary file 9 — Figure S8 [file 41416_2022_1764_MOESM9_ESM.tif]
