# Supplementary material for: Morpholino-driven blockade of Dkk-1 in osteosarcoma inhibits bone damage and tumour expansion by multiple mechanisms
Source: Br J Cancer. 2022 Mar 11;127(1):43–55. doi: 10.1038/s41416-022-01764-z (PMC9276700; doi:10.1038/s41416-022-01764-z)
Supplement: Supplementary file 10 — Supplemental materials [file 41416_2022_1764_MOESM10_ESM.docx]

**Supplementary Materials.**

Detailed Methods.

Supplemental figure legends:

Figure S1: Tumor expansion and Aldh1a1 expression.

Figure S2: Diagrammatic summary of interrelationships between GOTEA and HUB analysis datasets.

Figure S3: Histology of MOSJ-Dkk1 tumors.

Figure S4: Training and optimization of the neural net algorithm.

Figure S5: Histology of necrotic structures within MOSJ-Dkk1 tumors.

Figure S6: Summary of interrelationships between GOTEA and HUB analysis for *in vivo* HTS datasets.

Figure S7: DkkMo regulates genes associated with GO-terms related to cell attachment, migration and metastasis.

Figure S8: DkkMo perturbs motility in monolayer scratch assays.

Table S1: Differentially expressed genes in cultured MOSJ-Dkk1 cells treated with DkkMo.

Table S2: Differentially expressed genes in MOSJ-Dkk1 tumors treated with DkkMo and DRB.

Table S3: Differential expression of cancer stem cell markers in response to DkkMo.

**Detailed Methods.**

***Tissue Culture:*** Red fluorescent protein (RFP)-labeled MOSJ-Dkk1 and vector backbone control MOSJ-pLenti cells were generated as previously described (1). MOSJ-Dkk1 cells are a murine osteochondral sarcoma cell line (2) modified by the pLenti vector (Life Technologies, Carlsbad, CA) to constitutively express and secrete human Dkk-1 via the cytomegalovirus (CMV) promoter. The control line, MOSJ-pLenti, harbors the vector backbone with no cDNA cassette. Both cell lines were cultured in medium containing alpha-minimal-essential-medium (αMEM, Life Technologies) supplemented with 10% (v/v) fetal bovine serum (FBS, Atlanta Biologicals, Norcross, GA), 100 U.mL^-1^ penicillin & 100 µg.mL^-1^ streptomycin (Life Technologies) and 2 mM L-glutamine (Life Technologies). For expansion, cells were seeded at 500 cells per cm^2^ with media changes every two days. Adherent cells were recovered using 0.25% (w/v) trypsin/ethylene diamine tetra-acetic acid when a density of approximately 80-90% confluency was reached.

***IC50 determination:*** To determine DRB IC_50_ for the MOSJ-Dkk1 and MOSJ-pLenti lines, cells were seeded at 1000 cells per well in 12-well plates. DRB was added 24 hours after seeding. Cells were exposed to 8 doses of varying DRB concentration in log_10_ increments. Cell number was determined using a hemocytometer and the Ecotox software package (3) was used for IC_50_ calculations their comparisons.

***Scratch assays:*** 0.5 mm wide scratches were made to MOSK-Dkk1 monolayers using the edge of a standard cell scraper. Agents were added (DkkMo or scrMo at 25 μM) in standard culture media with changes every 2 days. Pictures at 10 x and 4 x were taken at random fields over the scratch every 12 hr for 7 days. Closure of the scratch was defined as a continuous monolayer indistinguishable from the periphery. For cell enumeration in the scratch zone, cells were counted in 2 randomly selected 4 x fields (2 fields, 5 cultures = 10 measurements) that had migrated to within the central 0.25 mm of the scratch zone (so as to exclude cells from the periphery).

***Enzyme linked immunosorbent assay (ELISA):*** Two-day conditioned media were collected and stored at -20◦C. For assays, media were thawed on ice, vortexed for 30 s then diluted (1 in 10 – 1 in 100) with PBS 0.05% (v/v) Tween20 with 1% (v/v) bovine serum albumin (BSA) then before performing ELISA. Human Dkk-1 ELISA was performed using a standard procedure recommended by the manufacturer (Duoset ELISA, R&D Systems).

***Quantitative RT-PCR (qRTPCR):*** The High Pure RNA isolation kit (Roche Diagnostics) was used for total RNA extraction from the cells. Copy DNA synthesis was performed using Superscript III kit (Life Technologies). TaqMan gene expression assays (Applied Biosystems) were used to carry out qRTPCR. APDH, MRPL19 and RPS18 were selected as reference genes (4-7). Reference genes were combined using geometric averaging as single internal control gene (5). Fold changes were calculated using the 2 ^−ΔΔCT^ method (8).

**In vivo *model of osteolytic OS:*** MOSJ-Dkk1 cells were employed in the OS xenograft model as described (1). Briefly, 1x10^6^ MOSJ-Dkk1 cells were administered to two month-old immune compromised nude mice (Foxn1^-^/Foxn1^-^, Jackson laboratories) in 50 μL of human plasma (Sigma, St. Louis, MO), with clotting activated facilitated by the addition of 50 μL of thromboplastin A (Sigma). The cells were injected medially, into the interosseous space between the tibia and fibula. Tumor expansion was monitored three times a week in live animals (n=10 per group) by detection of the RFP signal via live animal imaging using an IVIS Lumina III live animal imager (Perkin Elmer, Waltham, MA). For image analysis, photon flux from the tumors (cm^-2^ . s^-1^) was calculated for each mouse by using a fixed size circular region of interest (ROI) encompassing the back limbs of the mouse in both supine and prone position. Treatments were initiated in animals harboring tumors with an approximate volume of 15 mm^3^ (approximately 3 mm diameter) as estimated by fluorescence imaging. DRB (5 mg.kg^-1^), DKKMo (12.5 mg.kg^-1^) or a combination of the two was administered by intraperitoneal injection every 2 days. The dose of DRB was empirically established to be the maximum sub-lethal dose, and the concentration of DkkMo was recommended from previous studies safely utilizing morpholinos in mice (9, 10). During the treatment period, photon flux values were scaled to a comparable background value, then normalized to the value obtained at the first administration of treatment, ensuring all mice had an arbitrary starting signal of 0. Tumor growth rate was characterized by analyzing the delta photon flux over delta time. Slopes were compared using a mixed-model for repeated measures (MMRM) approach (11, 12). Group sizes were determined based on post-hoc power analyses using data from pilot studies and from our previous published work (1). Animals were randomized into groups upon arrival at the facility.

***Micro-CT (μCT) scanning:*** Once euthanized, mice were fixed by trans-cardiac perfusion. All samples (n=10 each group) were scanned using a SkysScan1275 system with the filtered (1.5 mm aluminum) beam set to 40 kV, 250 μA and image capture set to 11 μm resolution. Throughout the study, smoothing and beam hardening were fixed at 2% (smoothing kernel gaussian) and 25%, respectively. Ring artifact reduction and misalignment correction were adjusted manually to minimize scan artifacts. The dynamic range was set to between -1000 and 7519.5 HU for all reconstructions. Bone loss was calculated by comparison of volumes and bone mineral densities with the contralateral side. Bone mineral density (BMD) was measured using the attenuation coefﬁcient method with calcium hydroxyapatite phantoms (Bruker) as calibrants. The mean of three 0.1 mm^3^ regions corresponding to 25%, 37% and 50% of the total length of the bones beginning from the proximal end was measured. Half of the specimens were also contrast stained with iodine/potassium iodide (IKI) for two weeks (13), then rescanned under the same conditions to facilitate detection of soft tissues. These scans permitted accurate quantification of tumor volume. Specimens were randomized and blinded during microCT scanning of tumors.

***Bone deformation indices:*** 3D Slicer software (14) was used for bone volume and deformation measurements. Three dimensional renderings of the tumor-bearing and contralateral fibulae were constructed using the Otsu thresholding method (-1000 to 7519.5 HU), and the resultant bone model was then mirrored for registration. The mirrored model was computationally superimposed to contralateral bone model using a rigid algorithm to at least 5% accuracy. Models were further trimmed to the same anatomical landmarks. Model to model distances were calculated with the signed closest point approach. Bone deformation measurements were generated by quantifying the differences in coordinates between corresponding voxels in scans of the tumor-bearing versus contralateral images. The deformation analysis was done by quantile functional regression for regression analyses of distributions (15). In short, quantlet, a union set of elements was selected from the sample distribution to represent the whole distribution by lasso regression and cross-validated concordance. The quantlet from each sample was then fit to a quantlet space model using a Bayesian modeling approach. Distribution plots were analyzed for differences between one another using the using the Quantile Function on Scalar Regression Analysis for Distributional Data method (16). All code related to this process is available by contact with the corresponding author.

***High throughput sequencing:*** For proliferative cultures, MOSJ-Dkk1 cells were seeded at 500 cells per cm^2^ in 175 cm^2^ flasks and allowed to enter the logarithmic stage of growth after 6 days of culture with changes of media every 2 days. Groups (n=3) were no morpholino, 5 μM DkkMo, 5 μM scrambled morpholino control (scrMo) which was added 24 h after seeding. For the nutritional stress cohort, cells were seeded and cultured in the same manner, but exposed to 48 hr of confluence without media change for 4 days. Cells were collected after one week of treatment. In all cases, cells were recovered by trypsinization, washed in PBS, and stored as flash frozen pellet (1x10^6^ cells) in liquid nitrogen until mRNA extraction.

For tumor sequencing, mice were initially fixed by trans-cardiac perfusion and stored in phosphate-buffered 3% formaldehyde with 10 mM CaCl_2_ at 4^o^C. Four specimens from each group were randomly selected for RNA sequencing. Tumor/stroma was carefully dissected from bone and muscle tissue. Samples are sequenced by BGI Genomics (BGI Americas Corp. Cambridge, MA 02142, USA).

High throughput sequencing was performed on a NovaSeq 6000 system, recovering 150 million raw reads per sample in 150 bp paired-end format. The reads were aligned to the GRCm38.98 reference genome using HISAT2 v 2.1.0 (17). Gene counts were obtained by using SAMtools v.1.9 (18) and featureCounts (Subread v.2.0.0) (19, 20). The downstream differential expression analysis was performed in R (Core Team, 2020) (21) and with the DEseq2 package (22). Weighted correlation network analysis was done with the WGCNA package (23). Soft threshold was applied based on WGCNA manual suggestion. Hub genes were identified as top 10 correlated gene in gene significance cluster. Cytoscape (24) was used for network visualization. The functional analyses were performed with the Ingenuity Pathway Analysis Platform (IPA, Qiagen, Redwood City, CA). FPKM was calculated using DEseq2 as part of the IPA input. Gene ontology analysis was performed on PANTHER(25, 26).

All datasets are archived on the Gene expression Omnibus (GEO) database, Accession number: [GSE191143].

***Histology:*** Specimens were stored in 3% formaldehyde in PBS with 10 mM CaCl_2_ at 4^o^C. Decalcification was achieved with 1 M dibasic EDTA, pH 8.0. The solution was changed every 2 days. The samples were then dehydrated through increasing gradients of alcohols, followed with Sub-X clearing agent (Surgipath Medical Industries, Richmond, IL) and embedded in paraffin (Richard-Allan Scientific, San Diego, CA). Paraffin-embedded samples were cut to 9 µm sections. Sections were baked onto the slides at 60ºC for 1 h, followed by deparaffinized with Sub-X, and rehydration. For H&E staining, sections were stained in hematoxylin solution Gill number 3 (Sigma) and counterstained with 1% (w/v) eosin Y (Thermo Fisher) before clearing and dehydration. Masson’s trichrome staining was achieved using a commercially available kit (American Master Tech, Lodi, CA) following the manufacturer’s instructions.

***Neural net programming and utilization:*** A U-net architecture based Convolutional Neural Network (CNN) was built using TensorFlow v.2.3.0 (27, 28). The model was built with the Adam optimizer algorithm. One sample was selected by systematic random sampling from each treatment group and segmented manually. Tumor and necrotic tissue were segmented separately. In total, 2176 images were used for training (70% random assigned) and testing (19.5% random assigned) to construct the CNN model. Model accuracy (**FigS4**) was obtained by applying the model to the validating (10.5% random assigned) dataset. The validated CNN model then was used to segment the rest of the CT scans.

***Statistics:*** GraphPad Prism version 8.00 for Mac was used to plot data and carry out statistical analysis. Single means were compared using t-tests while multiple tests of means were carried out using one-way analysis of variance (ANOVA) and either Dunnett’s or Tukey’s post-test where appropriate. Specific statistical parameters are given in figure legends. The tumor growth rate is characterized by analysis of the slope of tumor fluorescence over time between groups. Slope changes compare to No treatment group were analyzed using a mixed-model for repeated measures (MMRM) approach. Restricted Maximum Likelihood (ReML) estimation was used. The MMRM model included the treatment group, time point, and treatment group-by-time point interaction as fixed effects; and animals within treatment group as random effects allowing induvial difference for tumor growth rate. An unstructured covariance structure was used to model the within-animal error. Distribution plots were analyzed for differences between one another using the using the Quantile Function on Scalar Regression Analysis for Distributional Data method (16).

**Supplementary Figure Legends.**

**Supplementary Figure 1:** Tumor expansion and Aldh1a1 expression. **Panel a:** Kaplan Meier survival plots for the calculation of the maximum tolerated DRB dose. **Panel b:** Plots of tumor expansion as a function of fold-change fluorescent intensity. Each line represents the mean fold-change fluorescence intensity. Individual points represent animals (n=8-9). Statistical analysis provided in Fig 4. **Panel c:**  Aldh1a1 transcription measured by qRT-PCR calculated as ΔCT in relation to aggregate measurements from a panel of standard reference genes (*left*) and as ΔΔCT versus MOSJ-pLenti measurements at day 2 of culture. **Panel d:** Aldh1a1 transcription measured by qRT-PCR calculated as ΔΔCT versus MOSJ-pLenti measurements at the appropriate day of culture. **Panel e:** Murine Aldh1a1, murine Dkk-1 and human Dkk-1 transcription in DRB treated and untreated MOSJ-Dkk1 cells at day 4 of culture measured by qRT-PCR calculated as ΔCT versus pooled reference genes. For panel c and d, comparisons are versus MOSJ-Dkk1 group at each day in culture. P values calculated by ANOVA with Dunnett’s post-test (n=3). padj<0.05 = *, padj<0.01= **, padj<0.005 = ***.

**Supplementary Figure 2:** Diagrammatic summary of interrelationships between GOTEA and HUB analysis datasets. The *orange* panel indicates all shared DE genes, the *blue* panel indicates hub genes from log phase cultures and the *pink* panel indicates hub genes from cultures under nutritional stress. Black lines indicate functional interconnections between DE genes.

**Supplementary Figure 3:** Histology of MOSJ-Dkk1 tumors. **Panel a-c:** H and E stained micrographs of untreated MOSJ-Dkk1 tumors (10x, *bar* = 200 μm, Panel a; 20x, *bar* = 100 μm Panel b; and 40x, *bar* = 50 μm, Panel c) indicating homogeneous and highly proliferative spindle-shaped tumor cells with extensive bone involvement (Panel a). **Panel d-f:** H and E stained composite images of untreated (Panel d), DRB treated (Panel e), DkkMo treated (Panel f), or combination treated (Panel g) tumors. Asterisks indicate necrotic foci, *bar* = 2 mm.

**Supplementary Figure 4:** Training and optimization of the neural net algorithm. **Panel a:** Comparison of the automated segmentation of tumor and necrotic foci (*predicted mask*) with manual segmentation (*true mask*) and the input image. **Panel b:** Examples of automated segmentation for each of the treatment groups. Input image (*left*), segmented tumor (*center*) and combined image (*right*) are presented. In the rightmost image, bone (*white*), soft tissue (*green*), tumor (*blue*) and foci (*cyan*) are indicated. **Panel c:** Plot of accuracy as a function of epoch during the training of the neural net. At the conclusion of training, accuracy was in excess of 80%.

**Supplementary Figure 5:** Histology of necrotic structures within MOSJ-Dkk1 tumors. **Panel a:** H and E (*above*) or Masson’s trichrome (*below*) stained composite micrographs of a combination treated tumor indicating collagenous infiltration of necrotic foci (blue stained areas with trichrome, *asterisks. Bar* = 2 mm). **Panel b:** H and E (*above*) or Masson’s trichrome (*below*) stained composite micrographs of necrotic lesions at higher power demonstrating that the collagen rich foci are essentially acellular (*bar* = 100 μm). **Panel c:** As Panel b, with indication of necrotic pyknosis (nuclear shrinkage) and nuclear fragmentation in cells adjacent to the foci.

**Supplementary Figure 6:** Summary of interrelationships between GOTEA and HUB analysis for *in vivo* HTS datasets. Categories in the *red* and *green* circles represent gene ontologies generated for the DkkMo versus NT and DRB versus combination comparisons respectively. The *blue* panel indicates hub genes generated form the DkkMo versus no treatment comparison, the *pink* panel indicates hub genes from the combination versus DRB comparison and the *orange* panel indicates shared DE genes. Black lines indicate functional interconnections between DE genes, hub genes and gene ontologies.

**Supplementary Figure S7:** GOTEA analysis using GO-terms responsible for cell attachment, migration and metastasis with differentially expressed genes from DkkMo versus NT and DRB versus combination comparisons.

**Supplementary Figure S8:** Monolayers of DkkMo cells were subjected to a 0.5 mm scratch and allowed to recover in the presence of 25 μM scrMo, DkkMo or no treatment for up to 80 hr (*bar* = 100 μm). **Panel a:** representative micrographs at low power. **Panel b:** Kaplan Meier curve plotting the rate of closure of scratches over time. **Panel c:** enumeration of cells present in the scratch zone in 2 randomly selected 10 x fields 24 hr after initiation of the scratch (from 5 replicate cultures), p-values calculated by ANOVA with Tukey post-test p<0.005 = ***.

1. Krause U, Ryan DM, Clough BH, Gregory CA. An unexpected role for a Wnt-inhibitor: Dickkopf-1 triggers a novel cancer survival mechanism through modulation of aldehyde-dehydrogenase-1 activity. Cell Death Dis. 2014;5:e1093.

2. Joliat MJ, Umeda S, Lyons BL, Lynes MA, Shultz LD. Establishment and characterization of a new osteogenic cell line (MOS-J) from a spontaneous C57BL/6J mouse osteosarcoma. In Vivo. 2002;16(4):223-8.

3. Hlina BL BO, Robinson CS, Dhiyebi H, Wilkie MP. Changes in the sensitivity of piscicide in an invasive species. North American Journal of Fisheries Management. 2019.

4. Abuna RPF, Oliveira FS, Ramos JIR, Lopes HB, Freitas GP, Souza ATP, et al. Selection of reference genes for quantitative real-time polymerase chain reaction studies in rat osteoblasts. J Cell Physiol. 2018;234(1):749-56.

5. Vandesompele J, De Preter K, Pattyn F, Poppe B, Van Roy N, De Paepe A, et al. Accurate normalization of real-time quantitative RT-PCR data by geometric averaging of multiple internal control genes. Genome Biol. 2002;3(7):RESEARCH0034.

6. Mandell JB, Lu F, Fisch M, Beumer JH, Guo J, Watters RJ, et al. Combination Therapy with Disulfiram, Copper, and Doxorubicin for Osteosarcoma: In Vitro Support for a Novel Drug Repurposing Strategy. Sarcoma. 2019;2019:1320201.

7. Rienzo M, Schiano C, Casamassimi A, Grimaldi V, Infante T, Napoli C. Identification of valid reference housekeeping genes for gene expression analysis in tumor neovascularization studies. Clin Transl Oncol. 2013;15(3):211-8.

8. Livak KJ, Schmittgen TD. Analysis of relative gene expression data using real-time quantitative PCR and the 2(-Delta Delta C(T)) Method. Methods. 2001;25(4):402-8.

9. Ferguson DP, Dangott LJ, Lightfoot JT. Lessons learned from vivo-morpholinos: How to avoid vivo-morpholino toxicity. Biotechniques. 2014;56(5):251-6.

10. Morcos PA, Li Y, Jiang S. Vivo-Morpholinos: a non-peptide transporter delivers Morpholinos into a wide array of mouse tissues. BioTechniques. 2008;45(6):613-4, 6, 8 passim.

11. Detry MA, Ma Y. Analyzing Repeated Measurements Using Mixed Models. JAMA. 2016;315(4):407-8.

12. Administration FaD. Statistical Approaches to Establishing Bioequivalence. Guidance for Industry. 2001.

13. Metscher BD. MicroCT for comparative morphology: simple staining methods allow high-contrast 3D imaging of diverse non-mineralized animal tissues. BMC Physiol. 2009;9:11.

14. Fedorov A, Beichel R, Kalpathy-Cramer J, Finet J, Fillion-Robin JC, Pujol S, et al. 3D Slicer as an image computing platform for the Quantitative Imaging Network. Magn Reson Imaging. 2012;30(9):1323-41.

15. Yang H, Baladandayuthapani V, Rao AU, Morris JS. Regression Analyses of Distributions using Quantile Functional Regression. arXiv preprint arXiv:181003496. 2018.

16. Yang H, Baladandayuthapani V, Rao AUK, Morris JS. Quantile Function on Scalar Regression Analysis for Distributional Data. J Am Stat Assoc. 2020;115(529):90-106.

17. Kim D, Langmead B, Salzberg SL. HISAT: a fast spliced aligner with low memory requirements. Nat Methods. 2015;12(4):357-60.

18. Li H, Handsaker B, Wysoker A, Fennell T, Ruan J, Homer N, et al. The Sequence Alignment/Map format and SAMtools. Bioinformatics. 2009;25(16):2078-9.

19. Liao Y, Smyth GK, Shi W. featureCounts: an efficient general purpose program for assigning sequence reads to genomic features. Bioinformatics. 2014;30(7):923-30.

20. Anders S, Pyl PT, Huber W. HTSeq--a Python framework to work with high-throughput sequencing data. Bioinformatics. 2015;31(2):166-9.

21. Team RC. R: A Language and Environment for Statistical Computin. 2020.

22. Love MI, Huber W, Anders S. Moderated estimation of fold change and dispersion for RNA-seq data with DESeq2. Genome Biol. 2014;15(12):550.

23. Langfelder P, Horvath S. WGCNA: an R package for weighted correlation network analysis. BMC Bioinformatics. 2008;9:559.

24. Shannon P, Markiel A, Ozier O, Baliga NS, Wang JT, Ramage D, et al. Cytoscape: a software environment for integrated models of biomolecular interaction networks. Genome Res. 2003;13(11):2498-504.

25. Thomas PD, Campbell MJ, Kejariwal A, Mi H, Karlak B, Daverman R, et al. PANTHER: a library of protein families and subfamilies indexed by function. Genome Res. 2003;13(9):2129-41.

26. Thomas PD, Kejariwal A, Guo N, Mi H, Campbell MJ, Muruganujan A, et al. Applications for protein sequence-function evolution data: mRNA/protein expression analysis and coding SNP scoring tools. Nucleic Acids Res. 2006;34(Web Server issue):W645-50.

27. Ronneberger O, Fischer P, Brox T, editors. U-Net: Convolutional Networks for Biomedical Image Segmentation2015; Cham: Springer International Publishing.

28. Abadi M, Agarwal A, Barham P, Brevdo E, Chen Z, Citro C, et al. Tensorflow: Large-scale machine learning on heterogeneous distributed systems. arXiv preprint arXiv:160304467. 2016.
